# Supplementary material for: Exploration of Novel Scaffolds Targeting Cytochrome b of Pyricularia oryzae
Source: Int J Mol Sci. 2023 Jan 31;24(3):2705. doi: 10.3390/ijms24032705 (PMC9917009; doi:10.3390/ijms24032705)
Supplement: Supplementary file 1 [file ijms-24-02705-s001.zip › ijms-2044459-supplementary.pdf]

Supporting information

Exploration of novel scaffolds targeting cytochrome *b* of *Pyricularia oryzae*

Cecilia Pinna[1†], Tommaso Laurenzi[2†], Fabio Forlani[1], Luca Palazzolo[2], Claire Beatrice Nolan[1], Michael S. Christodoulou[1], Paolo Cortesi[1], Andrea Pinto[1], Ivano Eberini[2,3*], Andrea Kunova[1*], and Sabrina Dallavalle[1]

Supplementary Table S1. Compounds selected after high-throughput virtual screening (HTVS) and interaction fingerprint clustering (SIFT)

Title	XPGscore [Kcal/mol]	mw	XPGscore/mw	Interactions
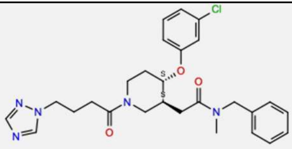 **BioDesign:122364**	-12.91	510.03	-0.025	-Glu273 H-bond
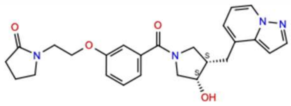 **BioDesign:88775**	-11.73	448.52	-0.026	-Glu273 H-bond<br>-Met296 H-bond
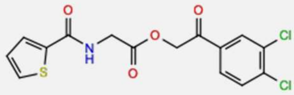 **Agrochem:9387**	-11.10	372.22	-0.029	-Glu273 H-bond<br>-Tyr280 π–π stacking
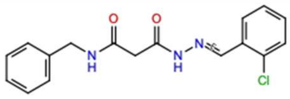 **Agrochem:6989_mod**	-10.08	329.79	-0.031	-Glu273 H-bond<br>-Tyr280 π–π stacking
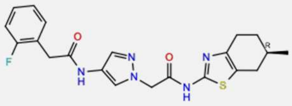 **Biodesign:7778**	-11.44	427.5	-0,027	-Glu273 H-bond<br>-Tyr275 H-bond<br>-Tyr280 π–π stacking
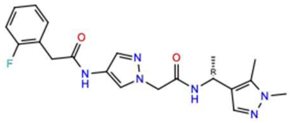 **Biodesign:7763**	-10.90	398.44	-0.027	-Glu273 H-bond<br>-Met125 H-bond<br>-Tyr279 π–π stacking<br>-Tyr280 π–π stacking
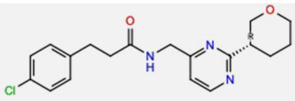 **Biodesign:61594**	-10.80	359.85	-0.030	-Tyr275 H-bond

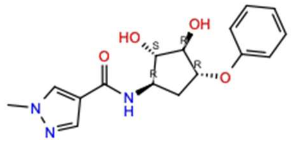 Biodesign:101550	-10.56	317.34	-0.033	-Glu273 H-bond<br>-Tyr275 H-bond
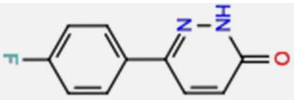 Biodesign:1678	-10.22	190.18	-0.054	-Tyr132 π–π stacking<br>-Tyr275 H-bond
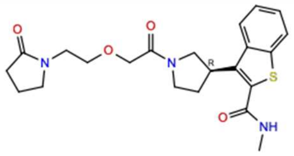 Biodesign:14185	-11.35	429.53	-0.026	-Glu273 H-bond
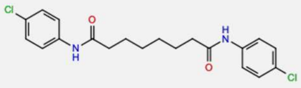 Agrochem:6151	-10.83	393.31	-0.028	-Glu273 H-bond<br>-Phe279 π–π stacking

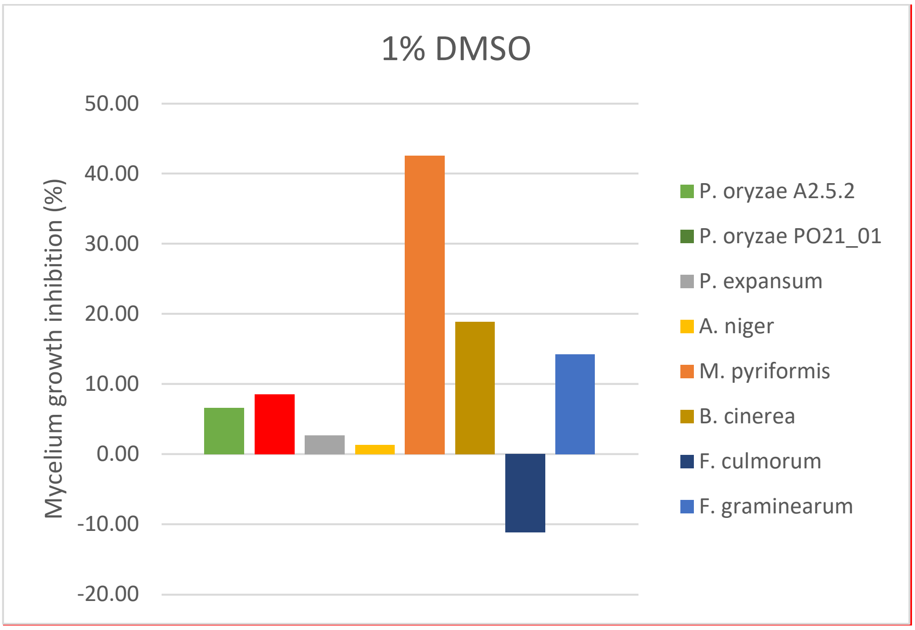

Supplementary Figure S1. Inhibition of mycelium growth of diverse fungi by 1% DMSO.

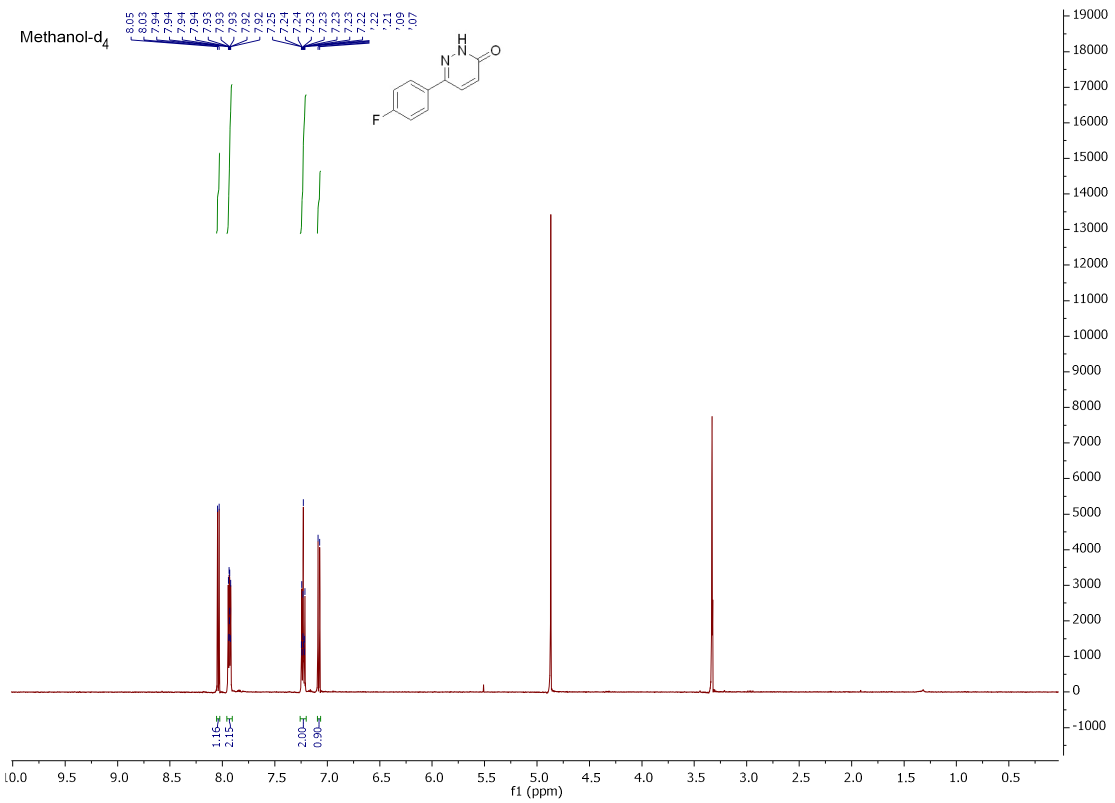

Supplementary Figure S2. ^{1}H-NMR **1a**.

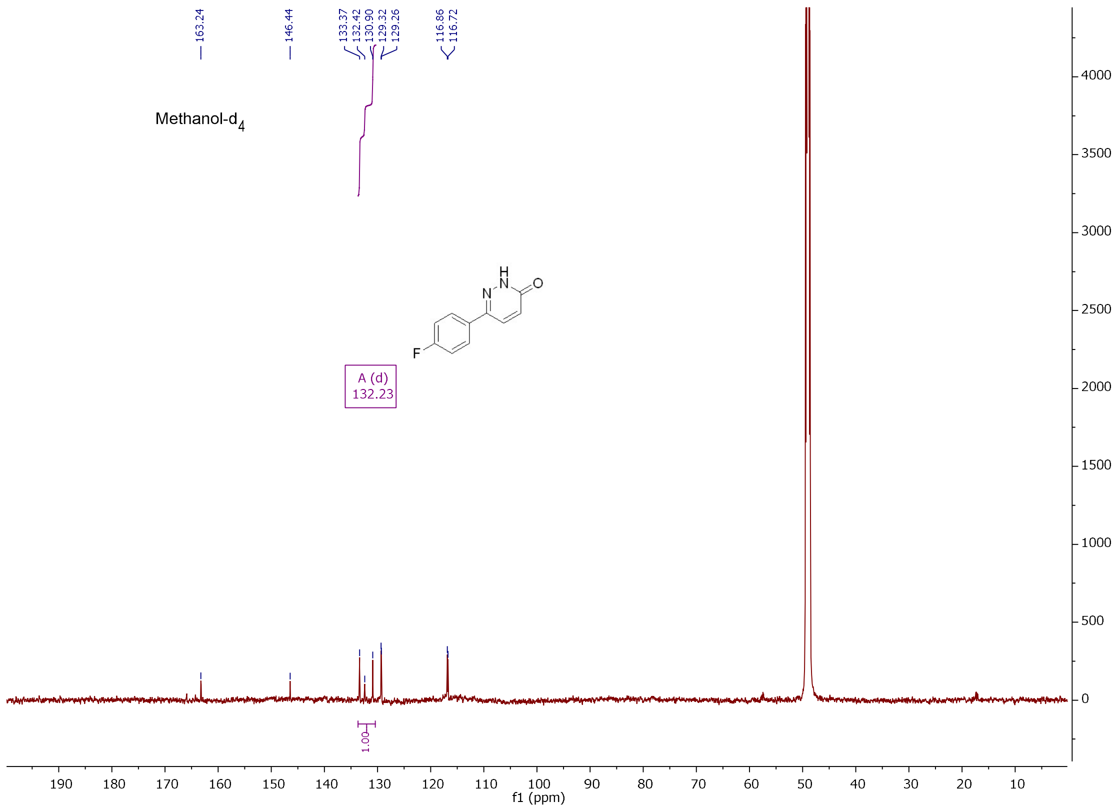

Supplementary Figure S3. ^{13}C-NMR **1a**.

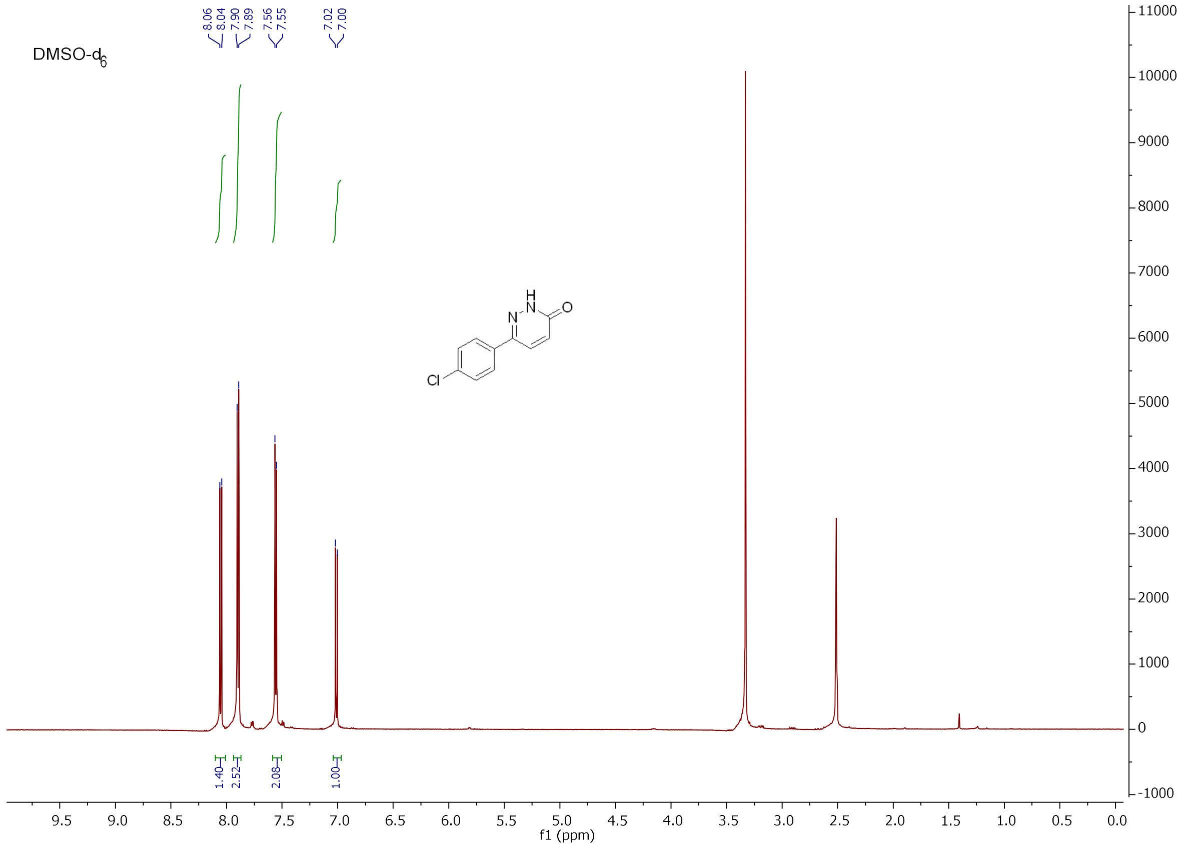

Supplementary Figure S4. ^{1}H-NMR **1b**.

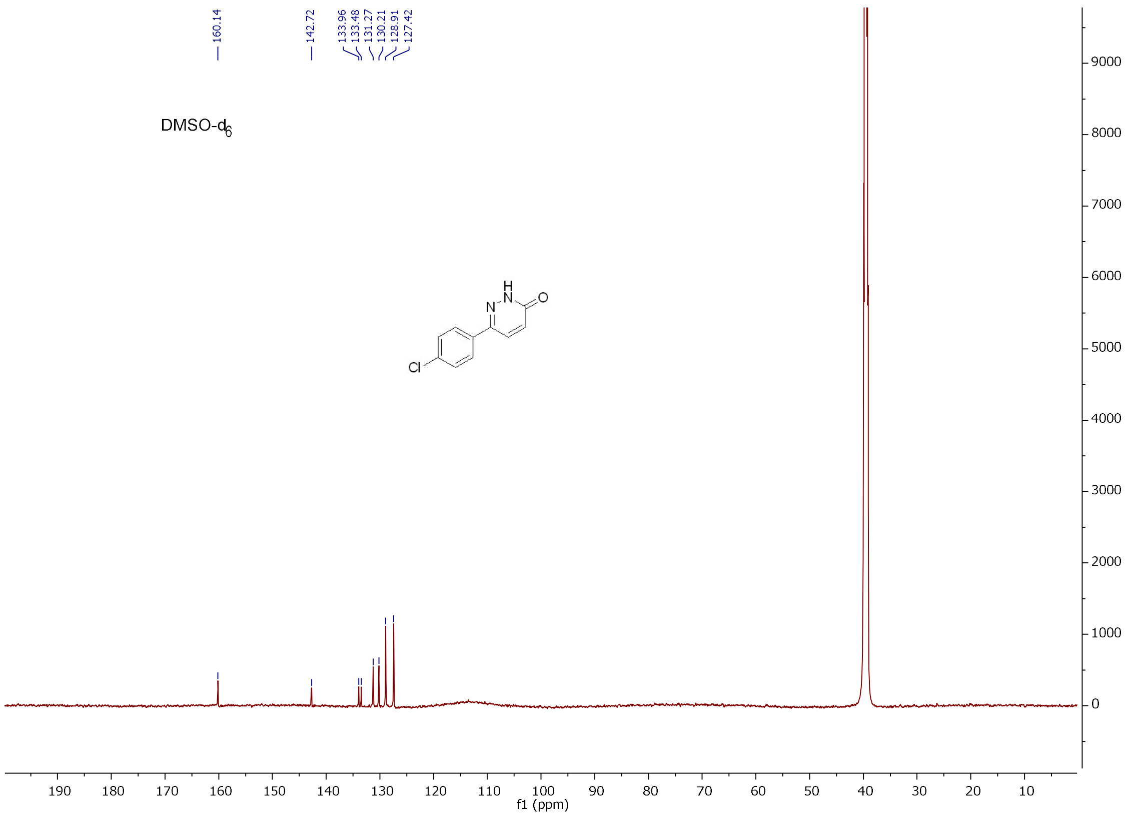

Supplementary Figure S5. ^{13}C-NMR **1b**.

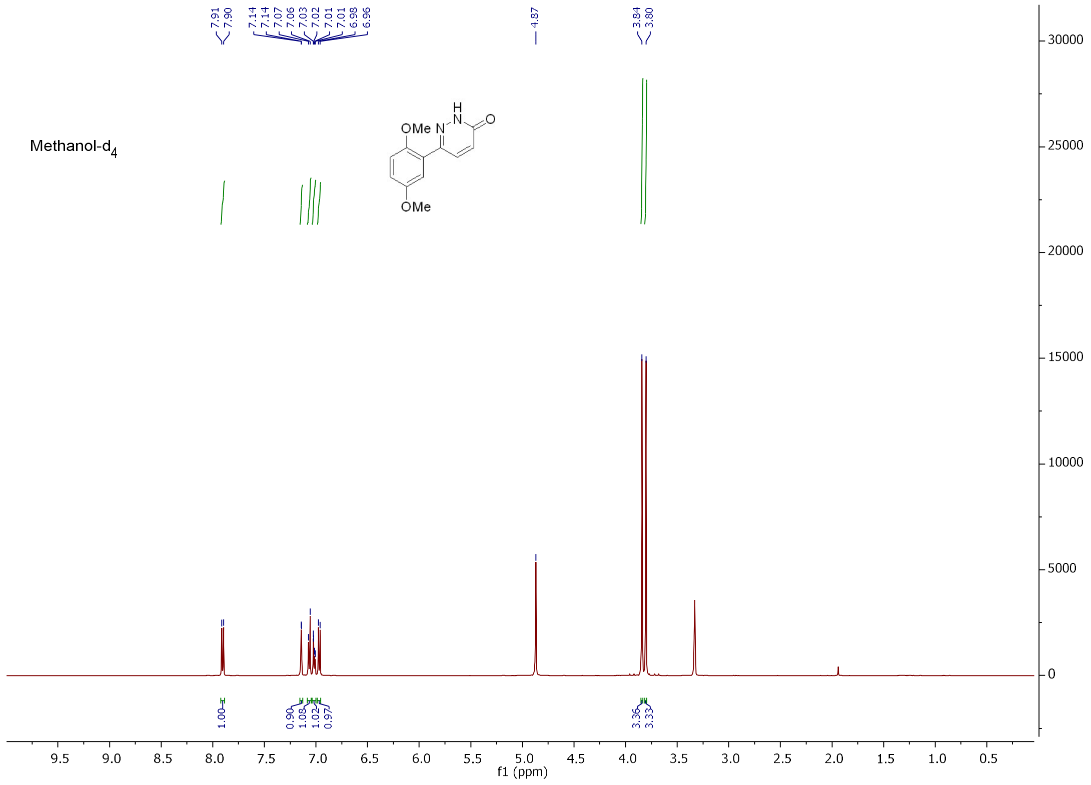

Supplementary Figure S6. [1]N-NMR **1c**.

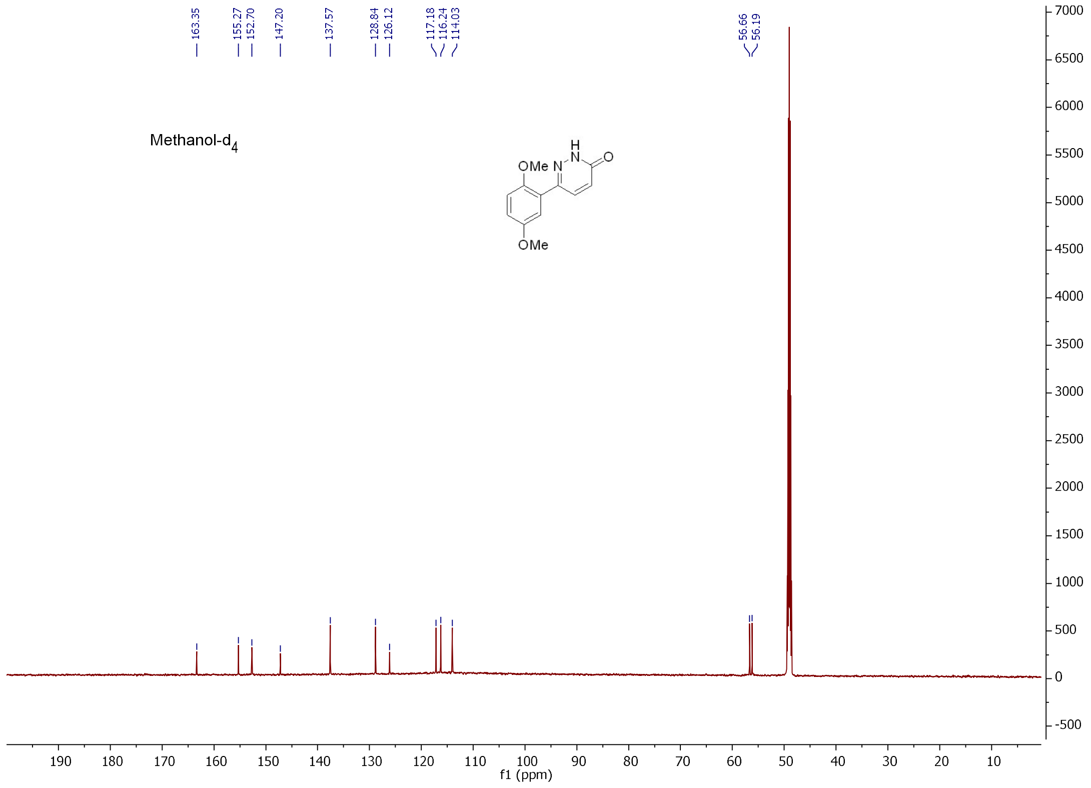

Supplementary Figure S7. ^{13}C-NMR **1c**.

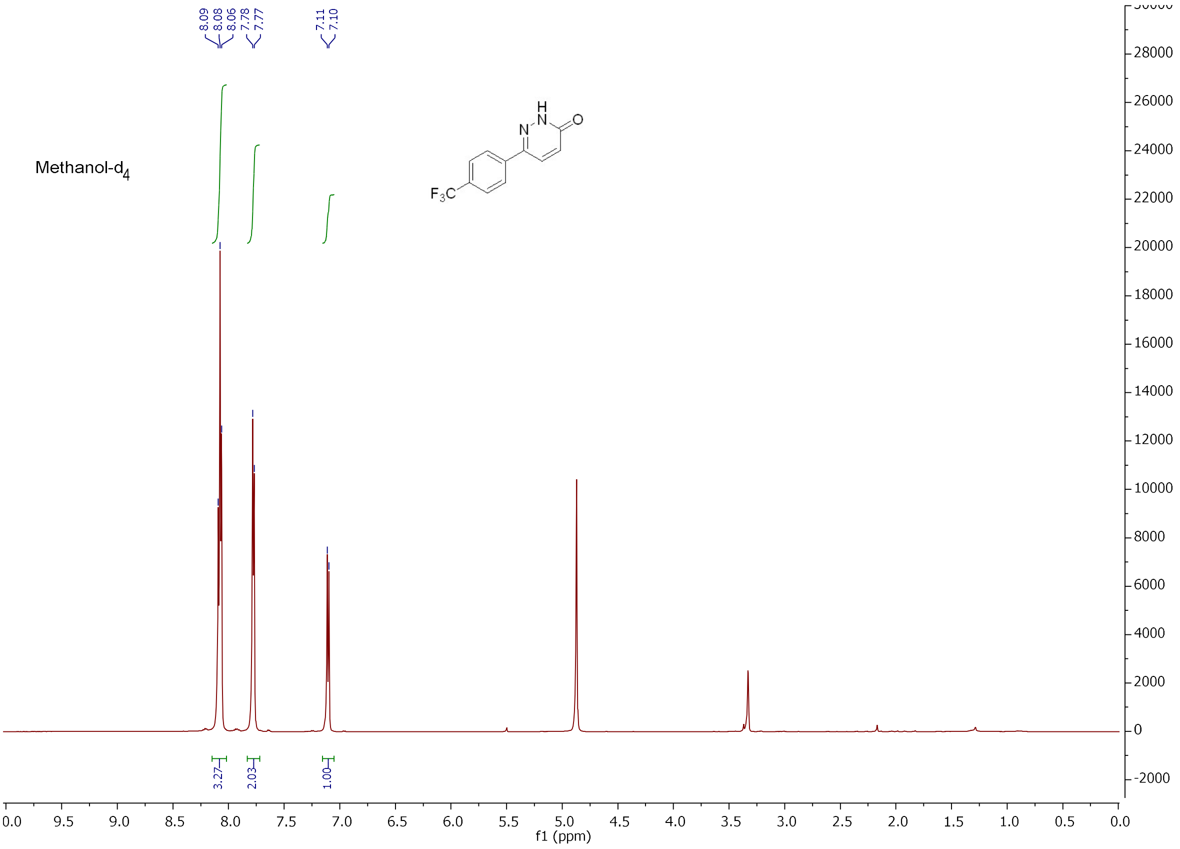

Supplementary Figure S8. ^{1}N-NMR **1d**.

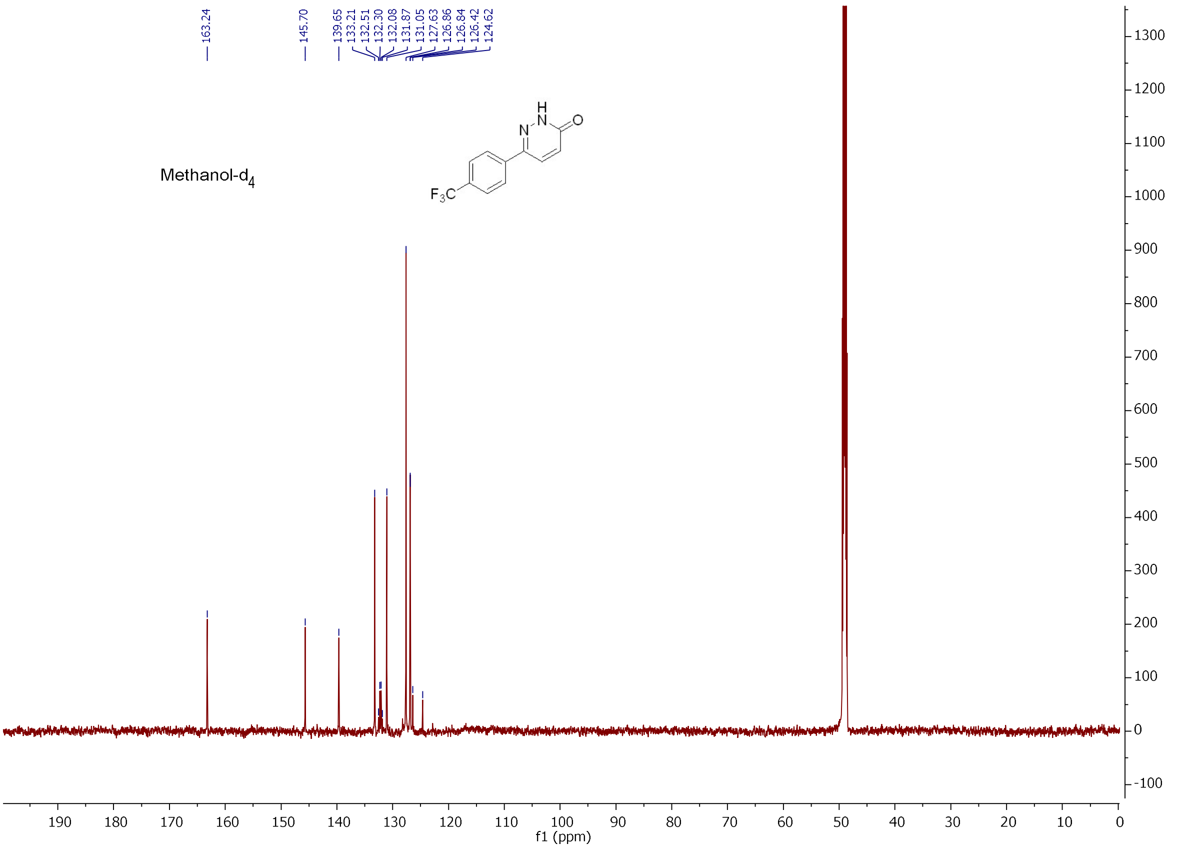

Supplementary Figure S9. ^{13}C-NMR **1d**.

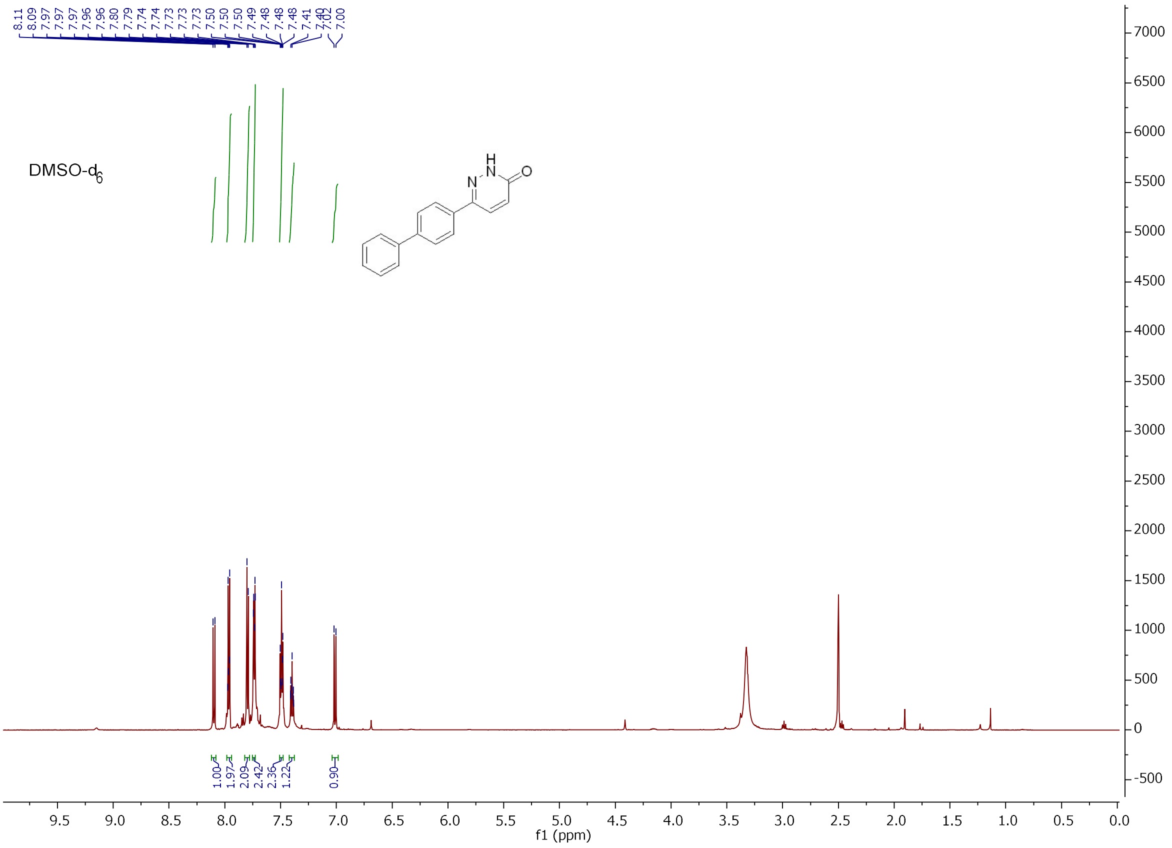

Supplementary Figure S10. ^{1}N-NMR **1e**.

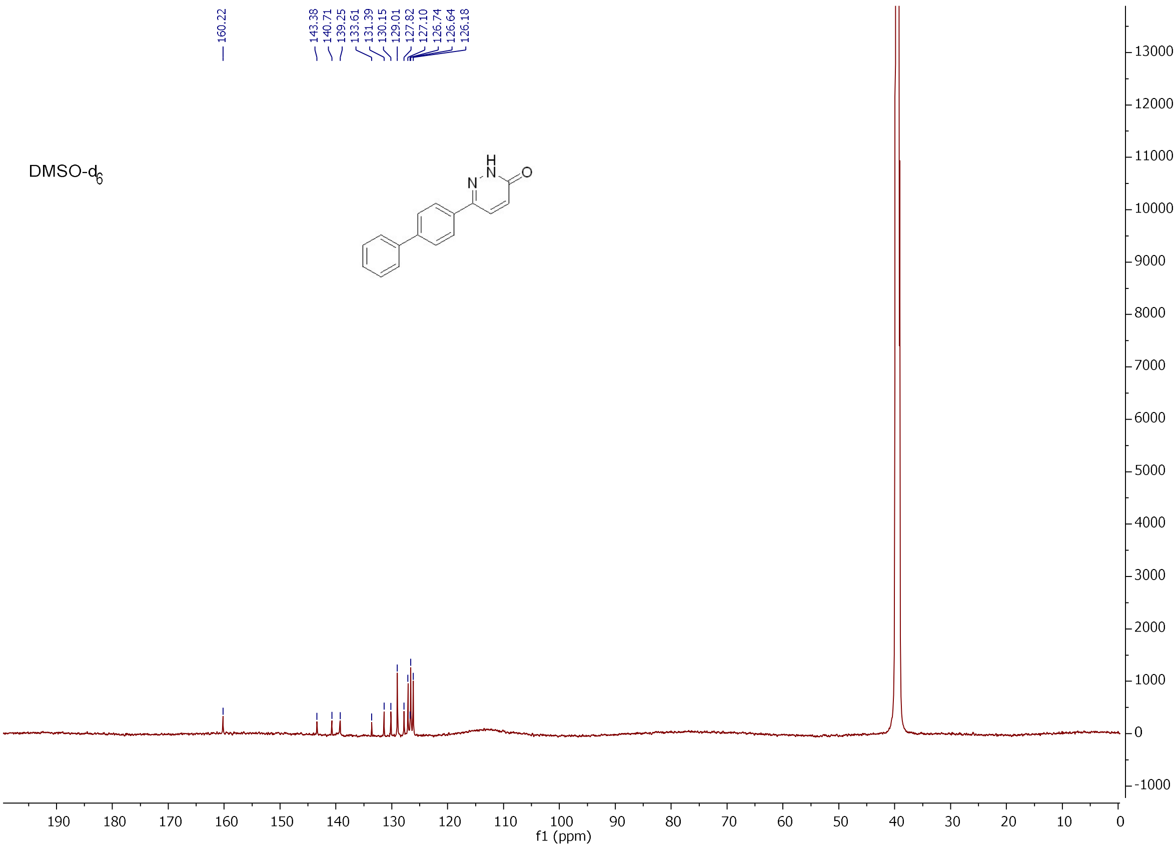

Supplementary Figure S11. ^{13}C-NMR **1e**.

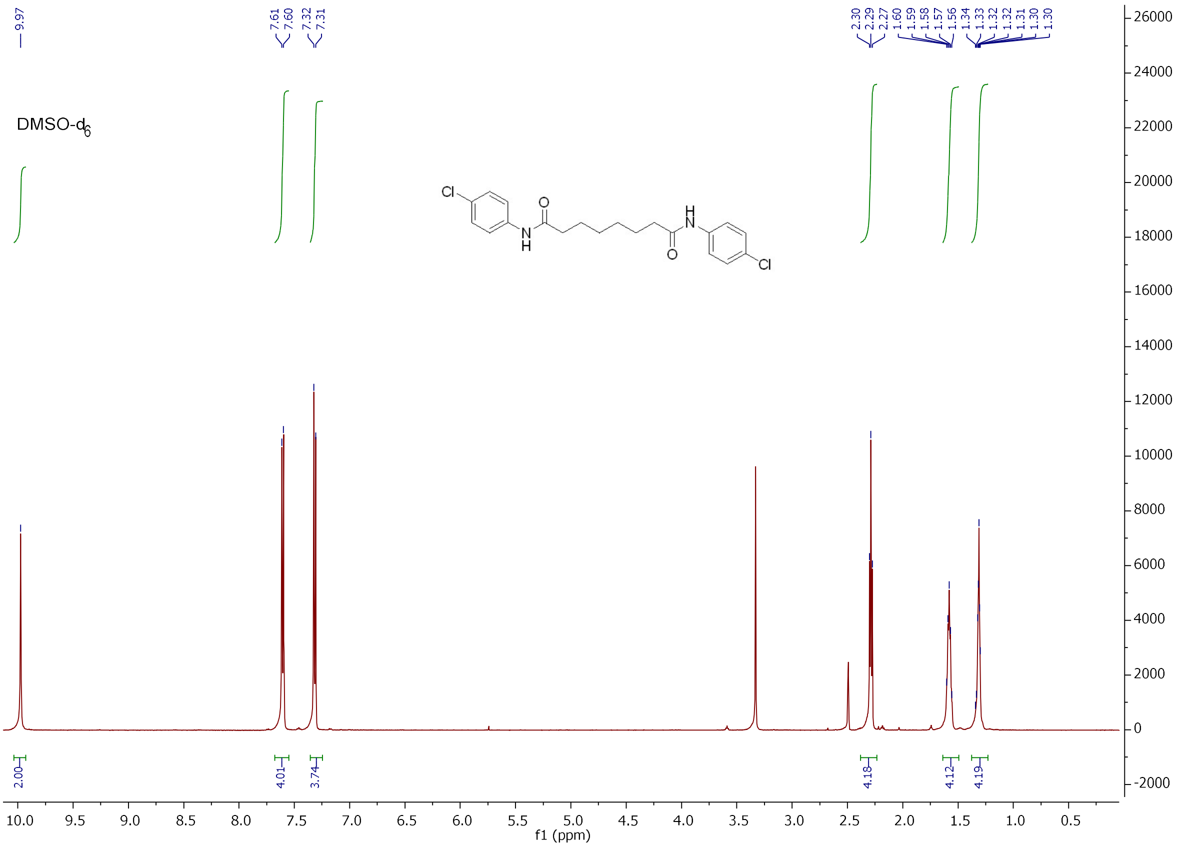

Supplementary Figure S12. [1]N-NMR **2a**.

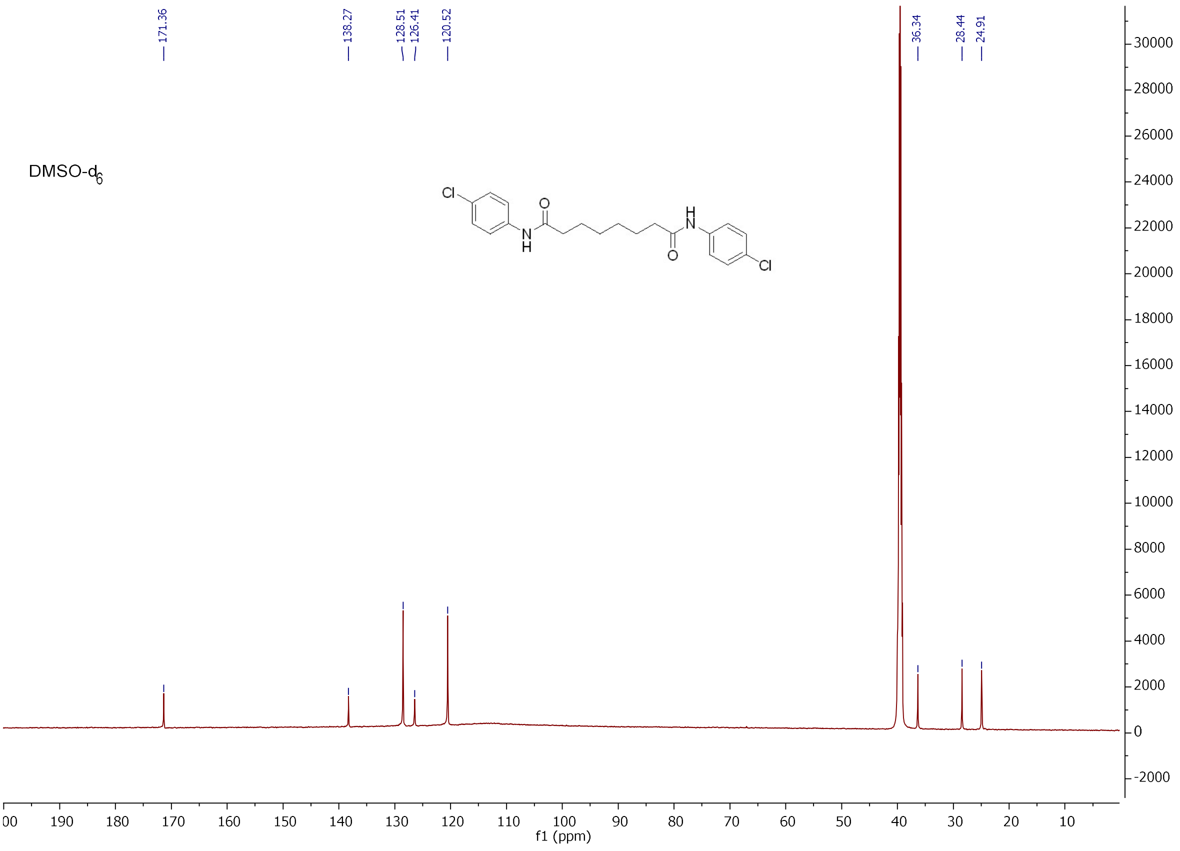

Supplementary Figure S13. ^{13}C-NMR **2a**.

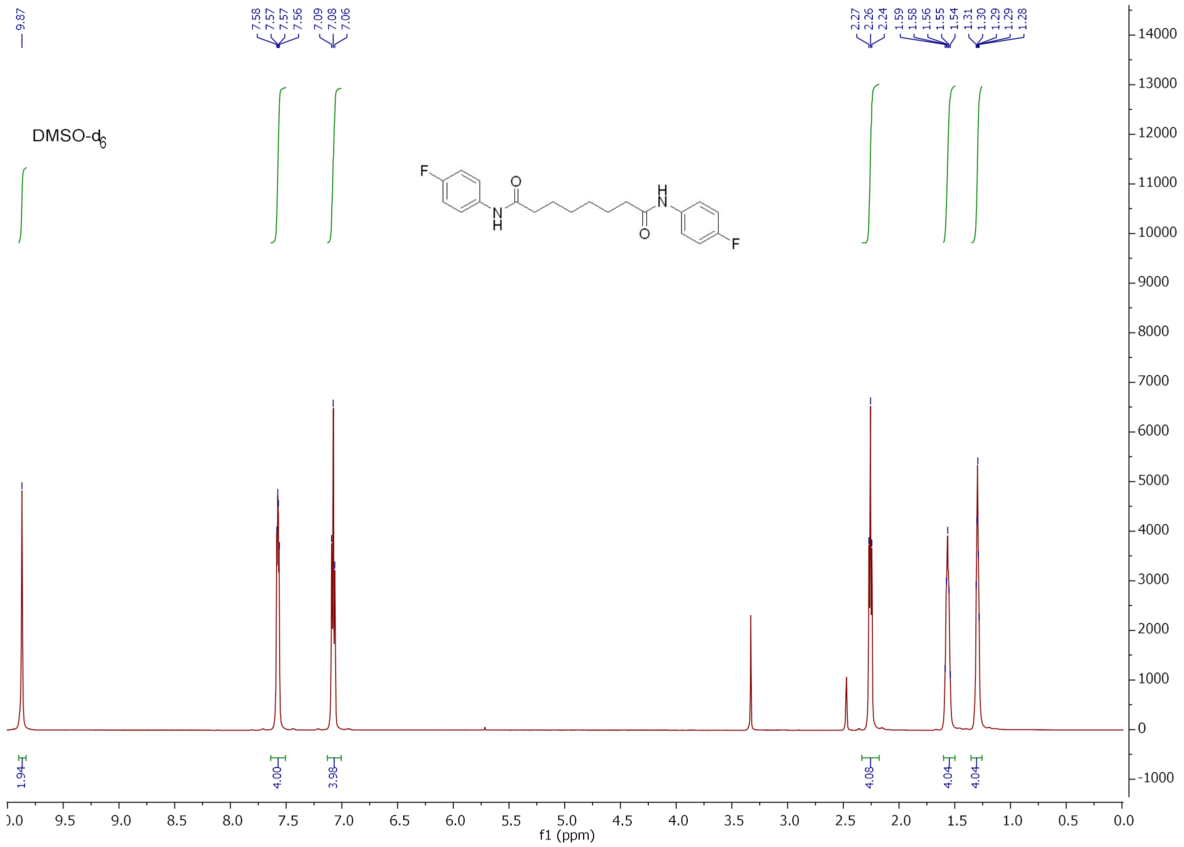

Supplementary Figure S14. ^{1}N-NMR **2b**.

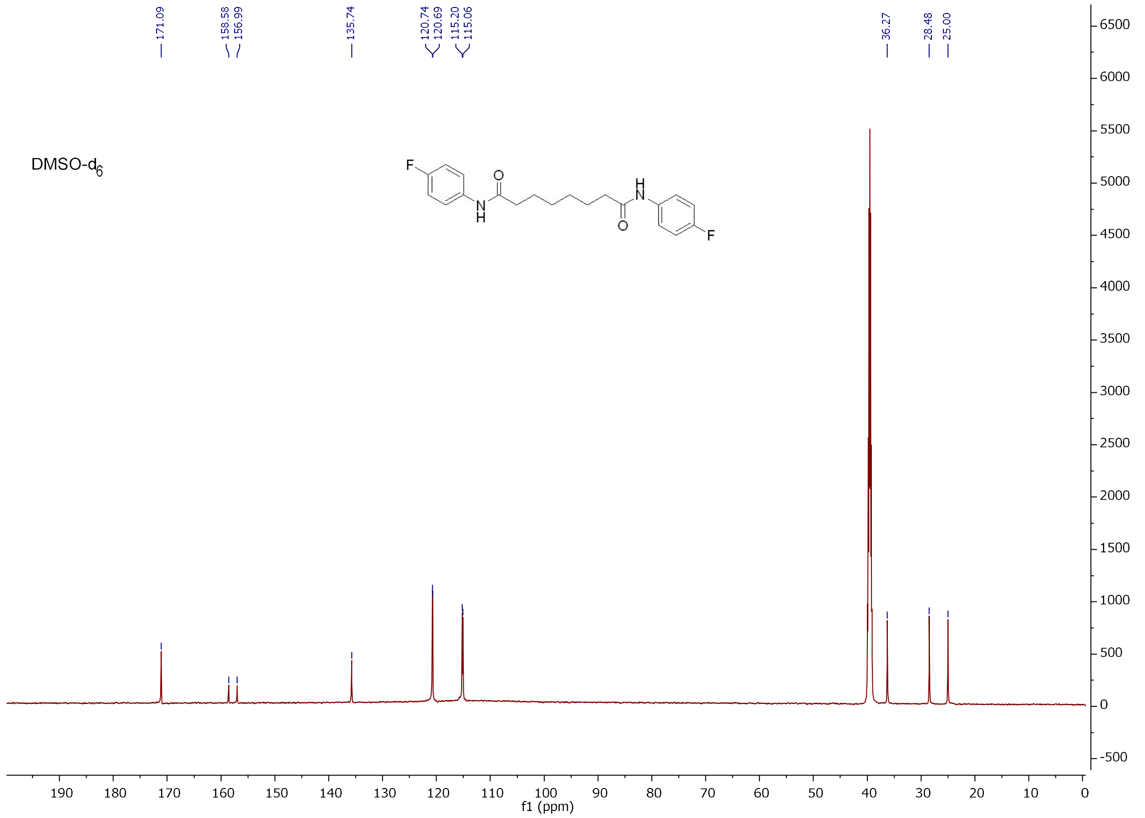

Supplementary Figure S15. ^{13}C-NMR **2b**.

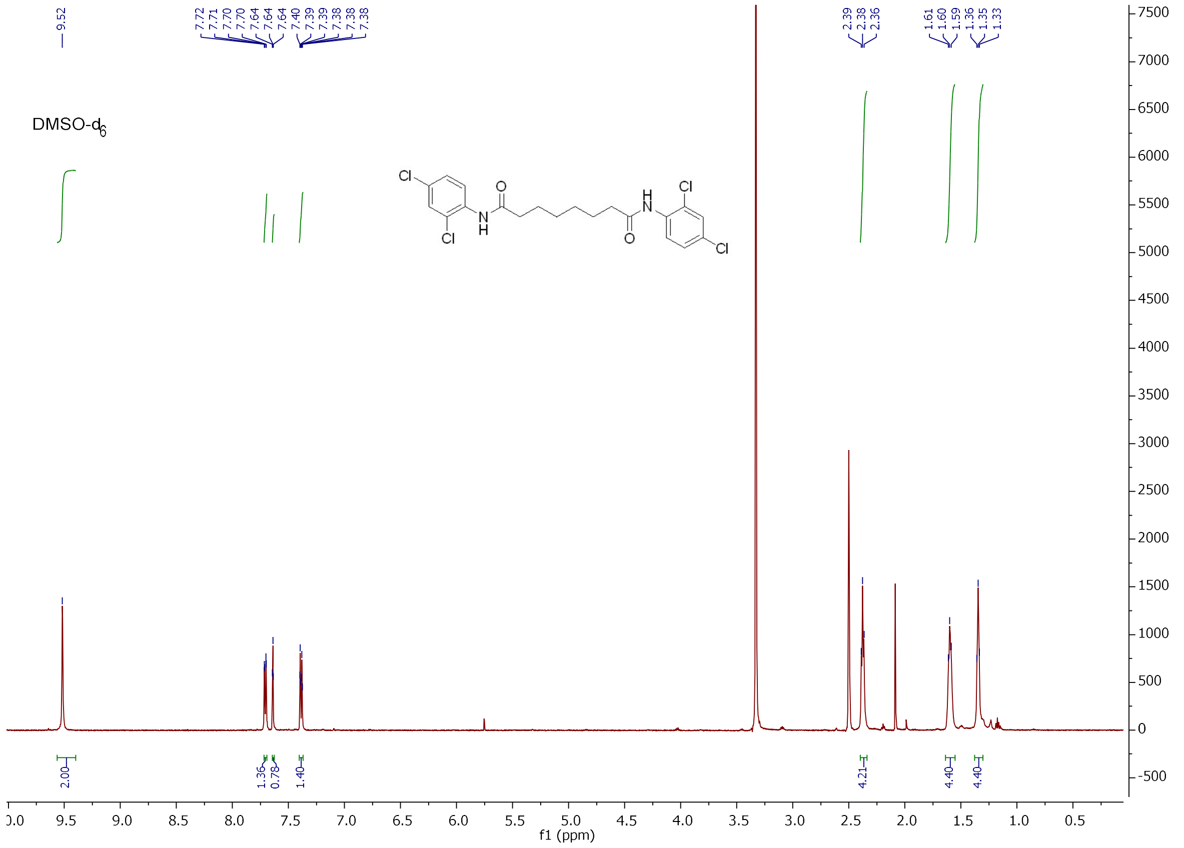

Supplementary Figure S16. ¹N-NMR **2c**.

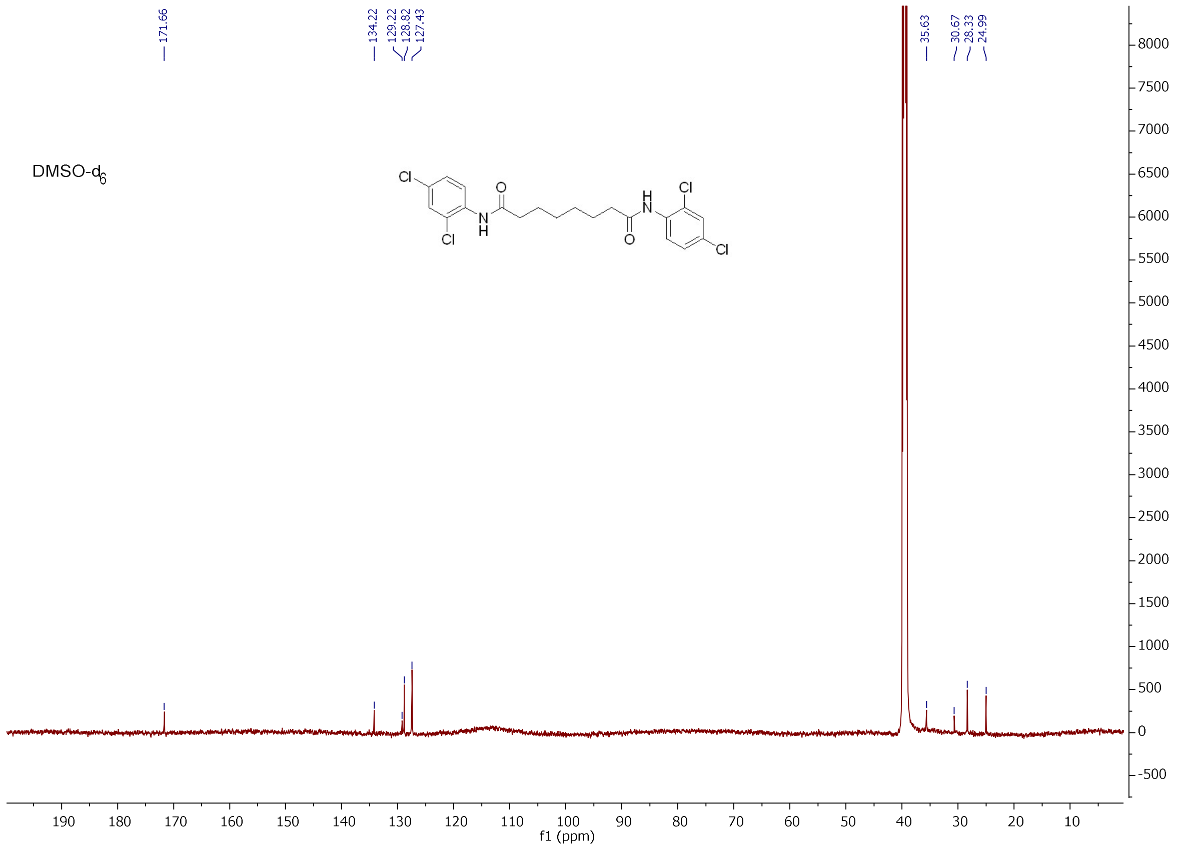

Supplementary Figure S17. ¹³C-NMR **2c**.

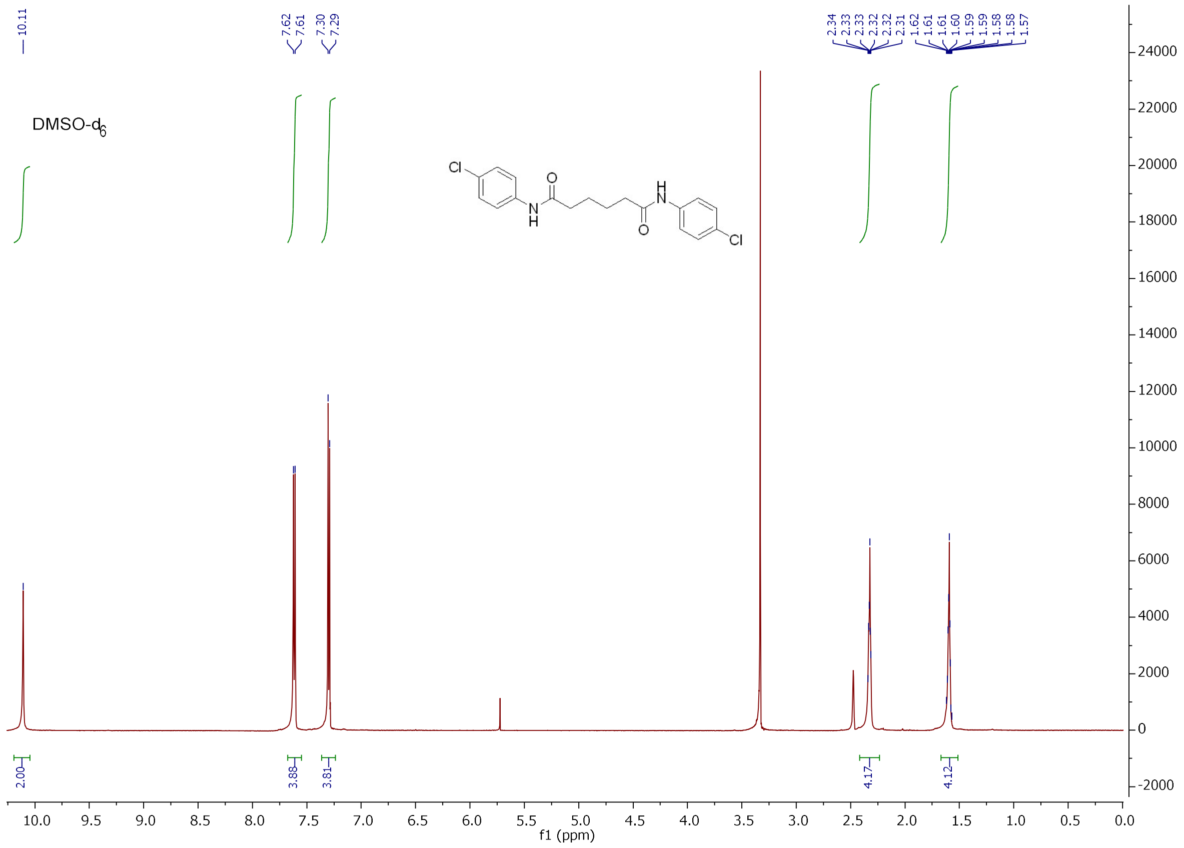

Supplementary Figure S18. [1]N-NMR **2d**.

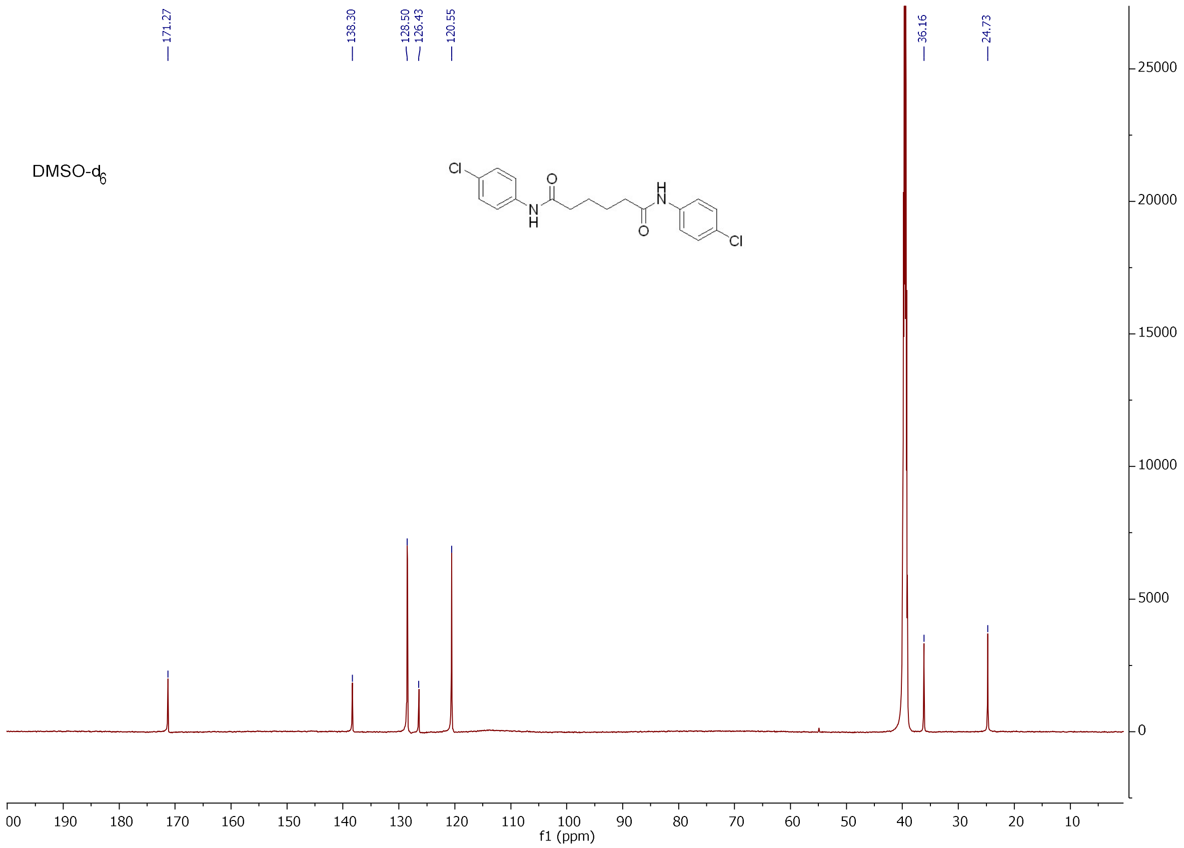

Supplementary Figure S19. ^{13}C-NMR **2d**.

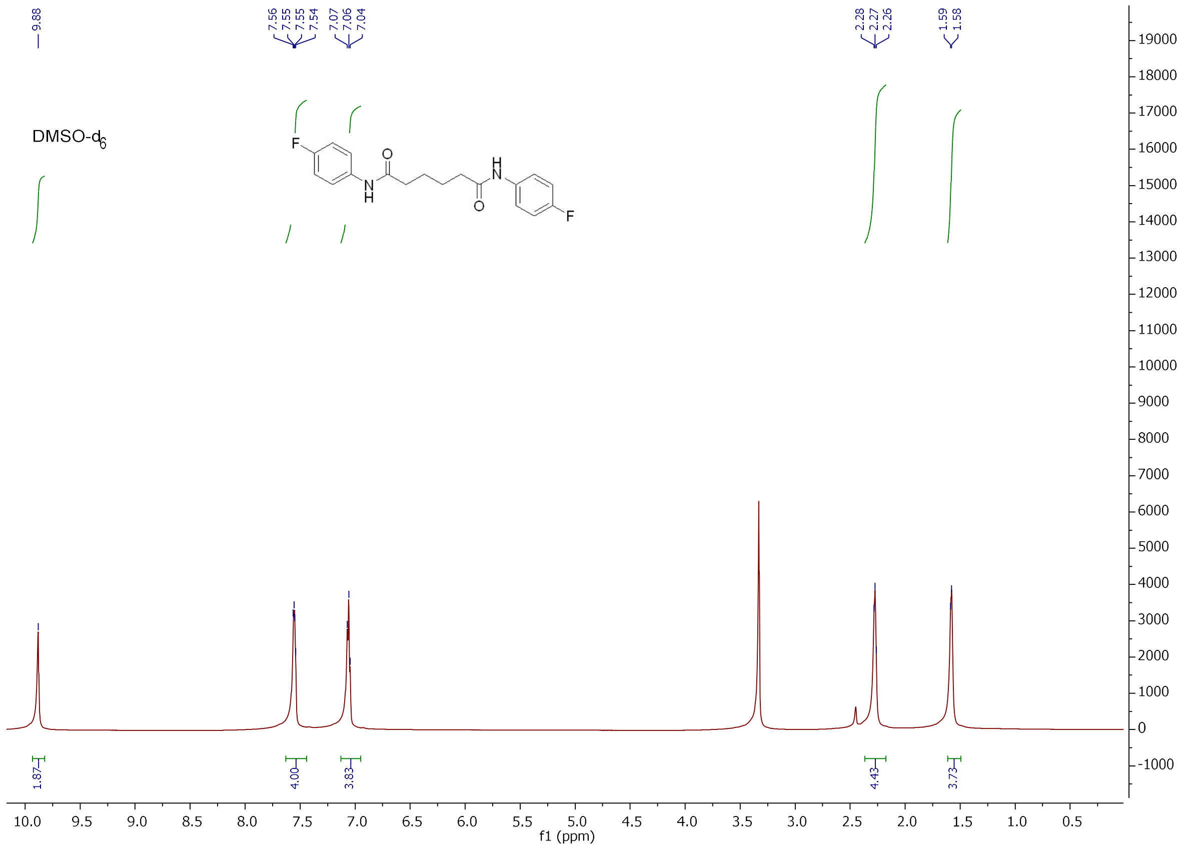

Supplementary Figure S20. ^{1}N-NMR **2e**.

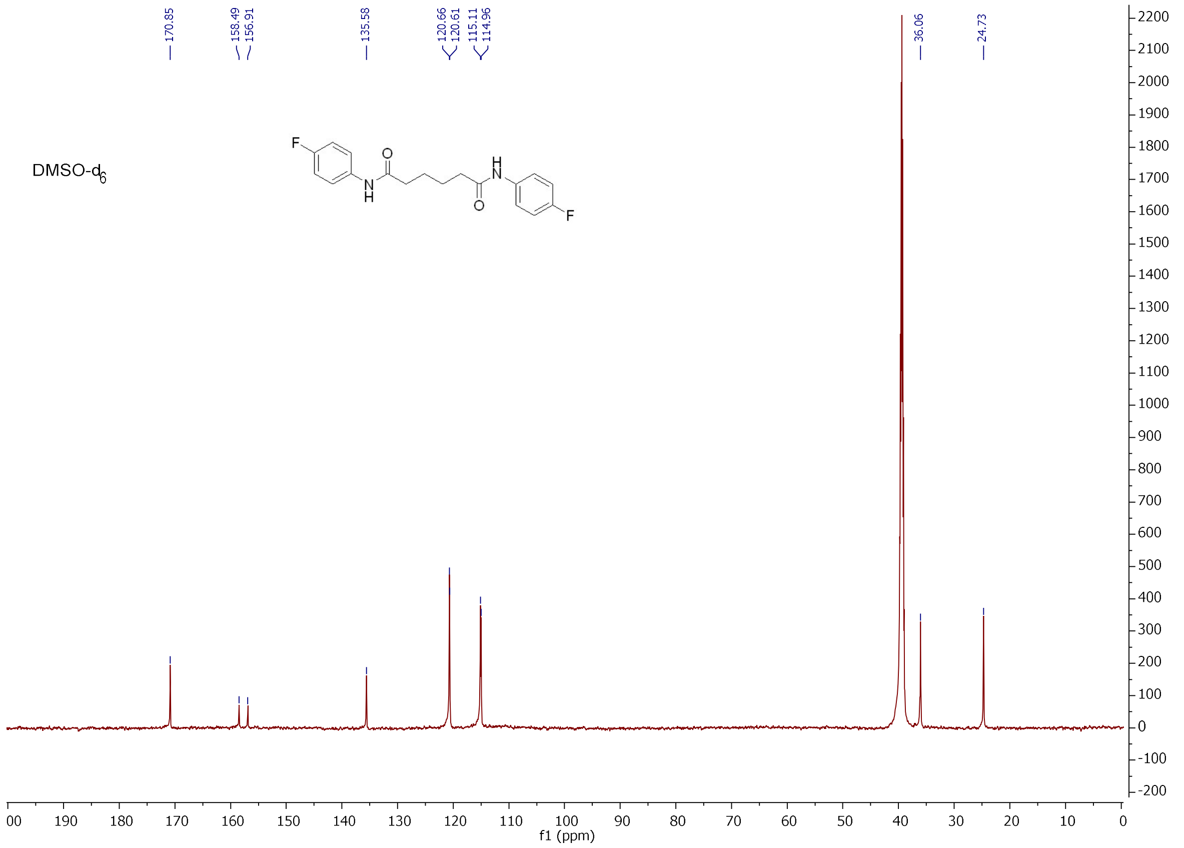

Supplementary Figure S21. ^{13}C-NMR **2e**.

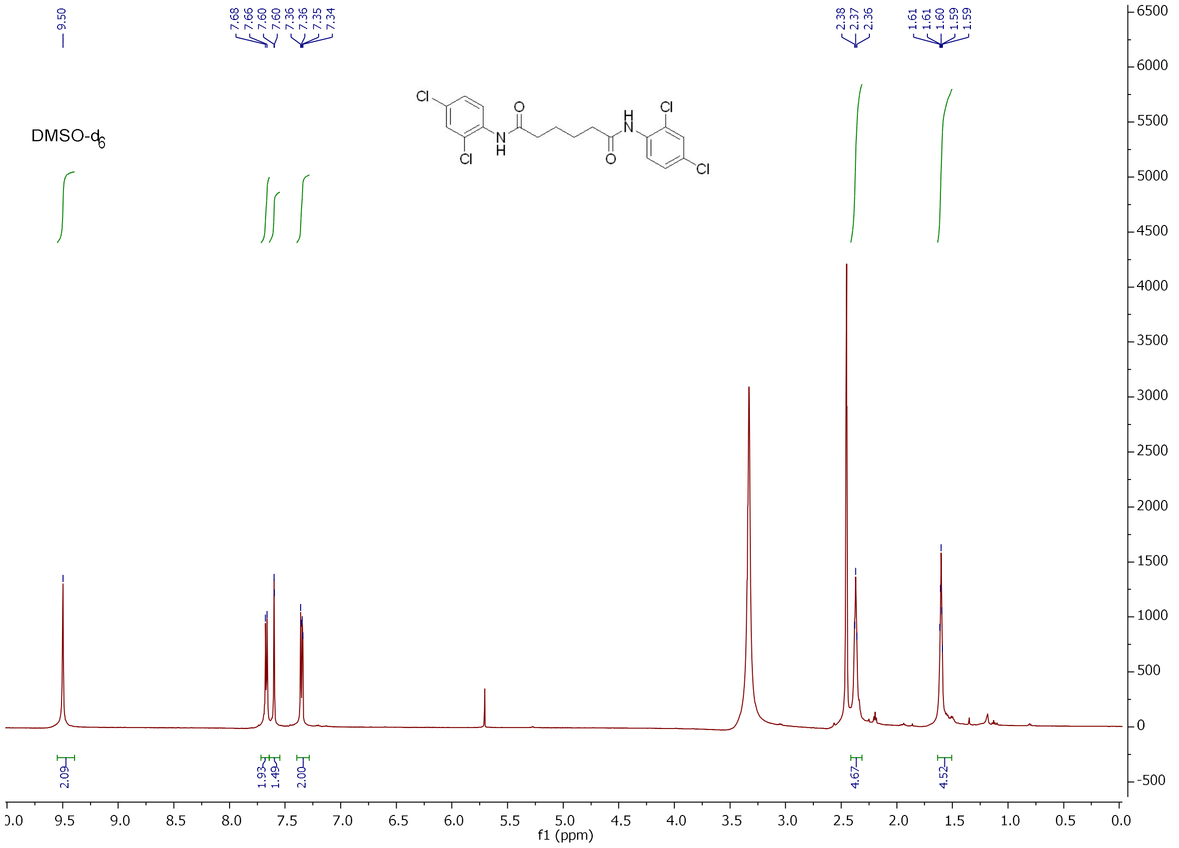

Supplementary Figure S22. [1]N-NMR **2f**.

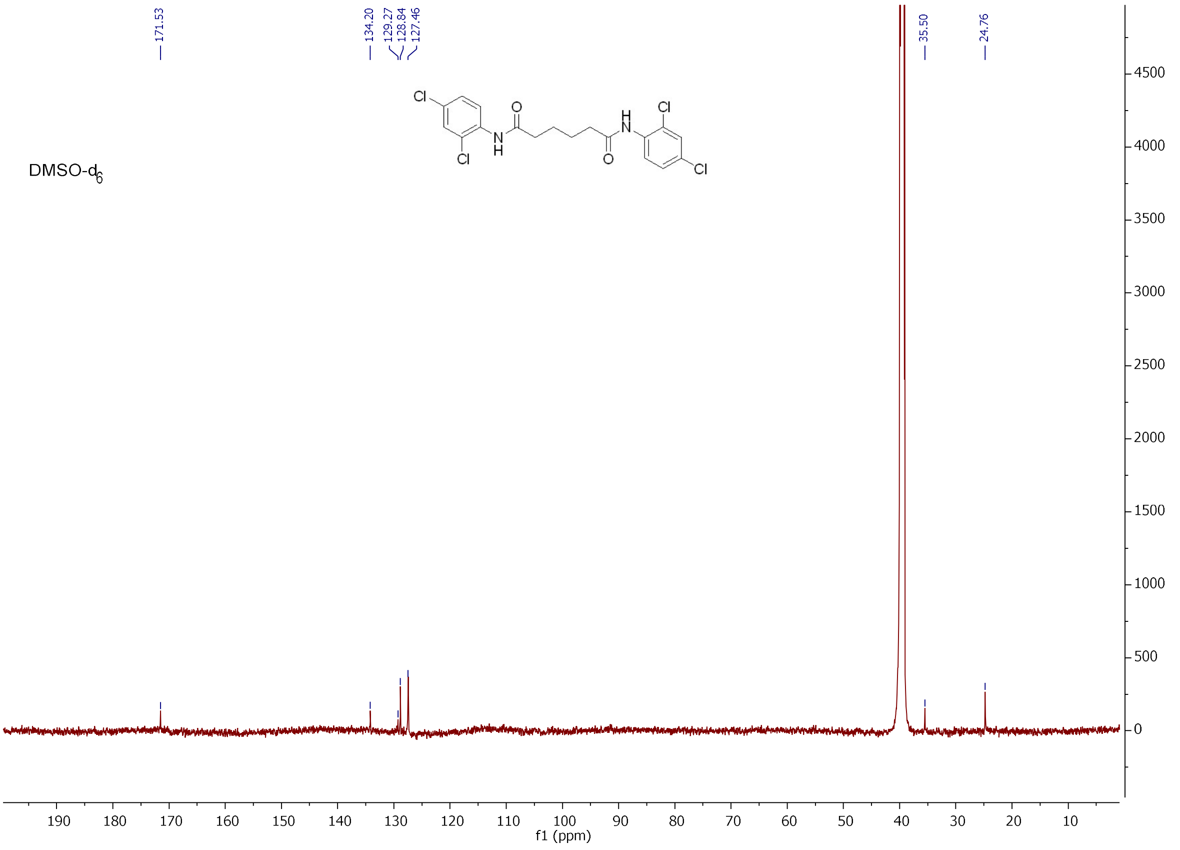

Supplementary Figure S23. ^{13}C-NMR **2f**.

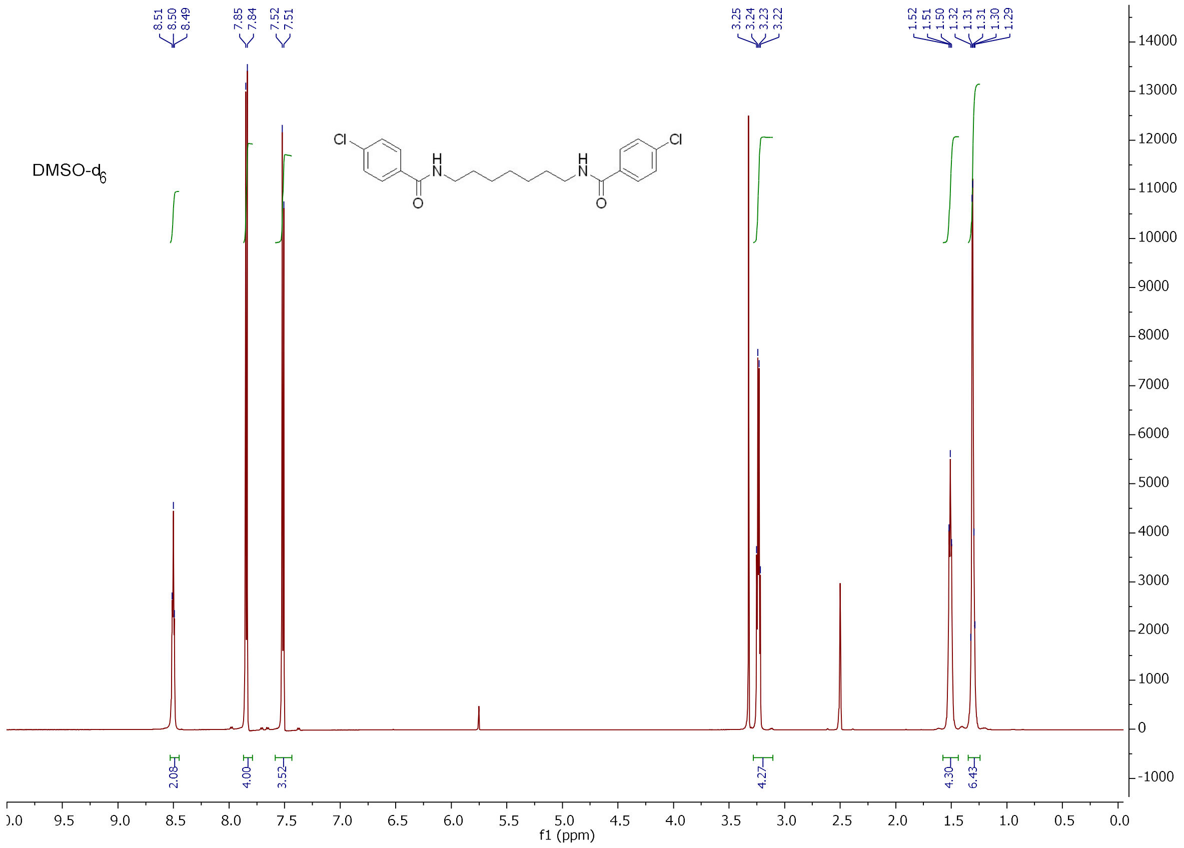

Supplementary Figure S24. [1]N-NMR **5a**.

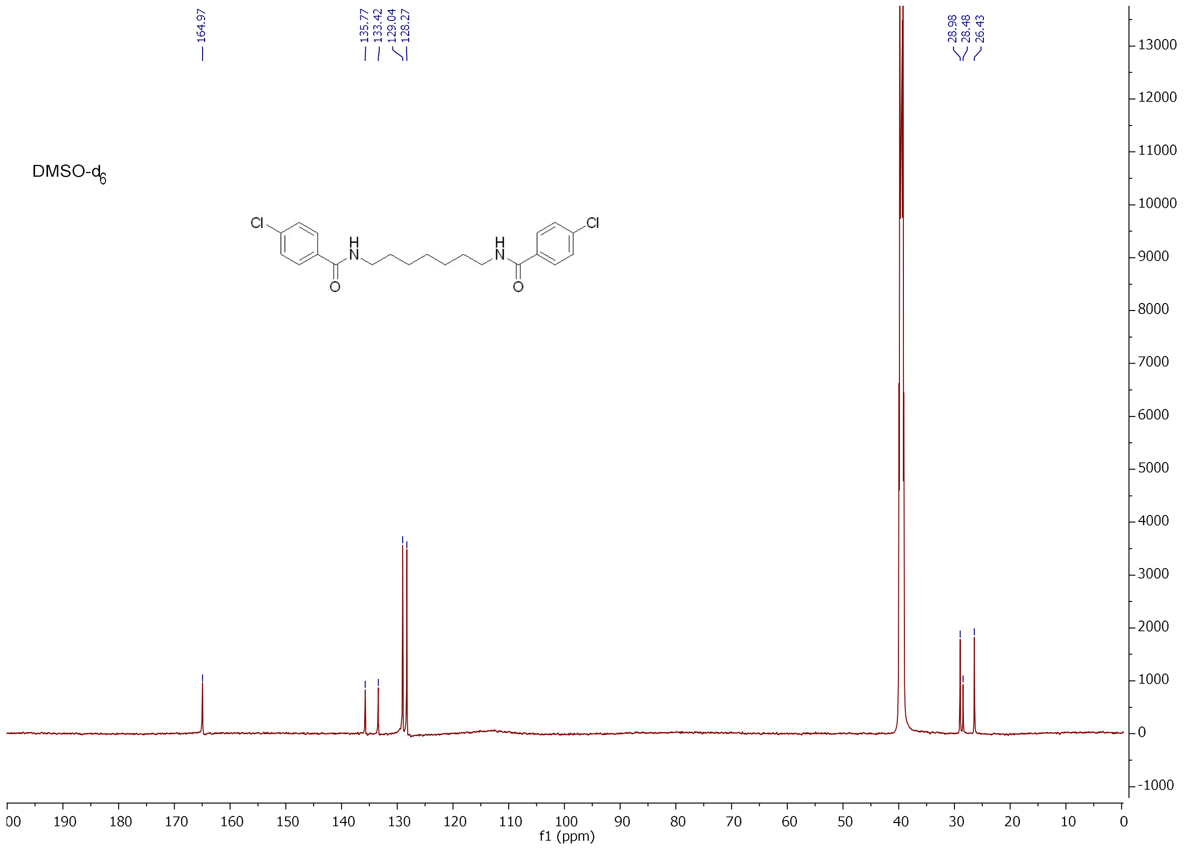

Supplementary Figure S25. ^{13}C-NMR **5a**.

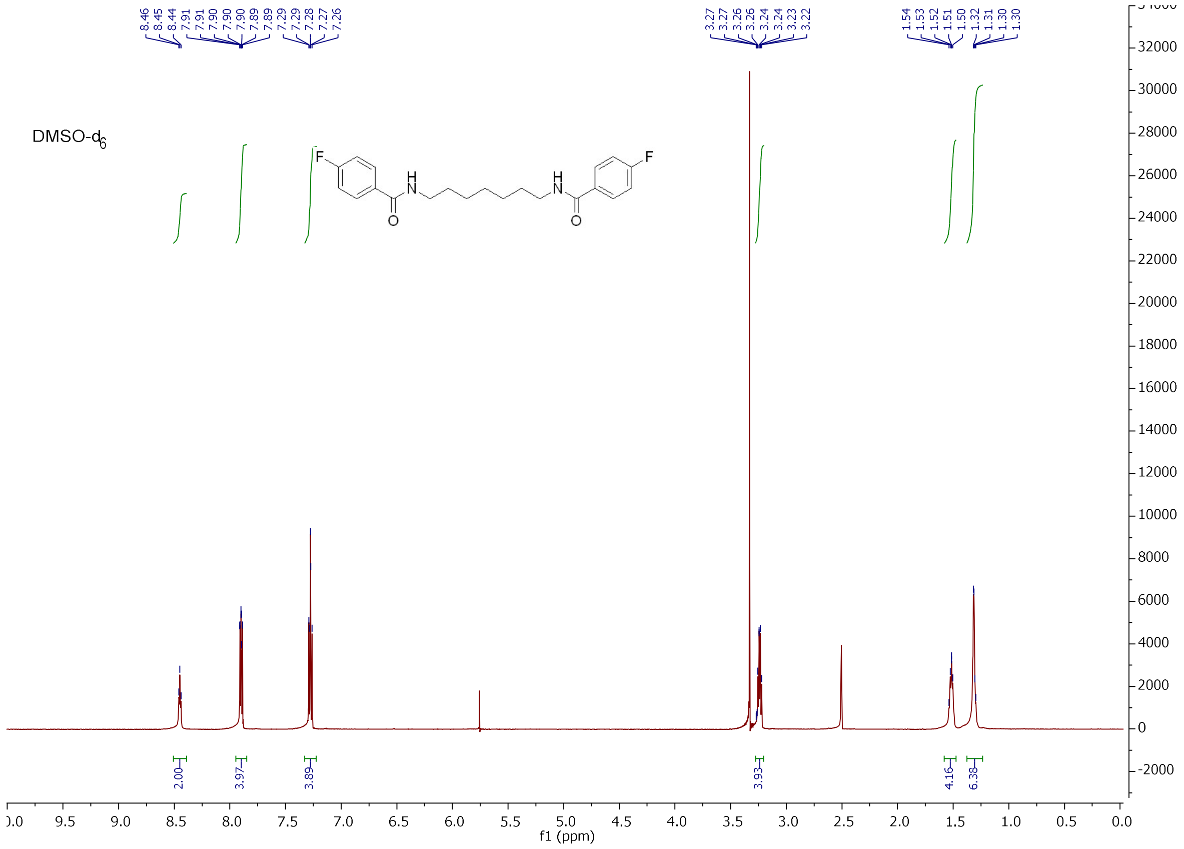

Supplementary Figure S26. ^{1}N-NMR **5b**.

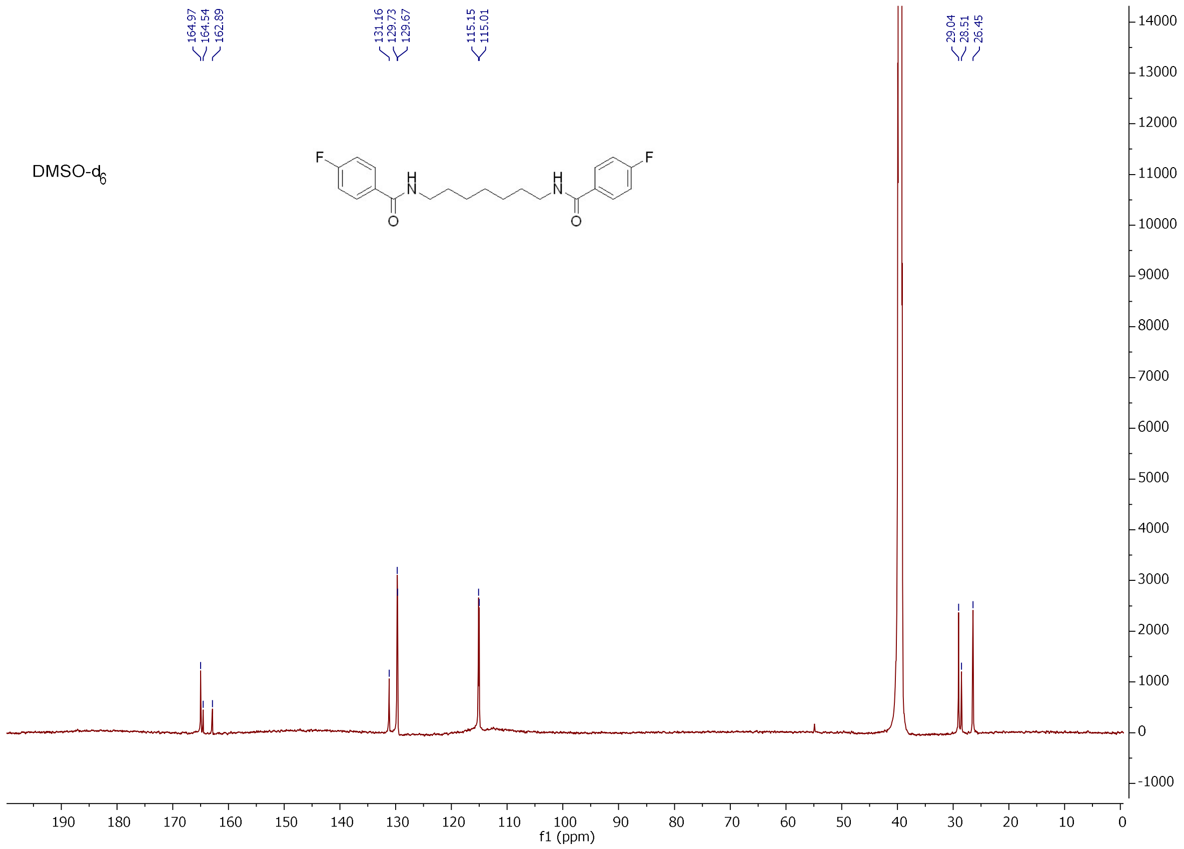

Supplementary Figure S27. ^{13}C-NMR **5b**.

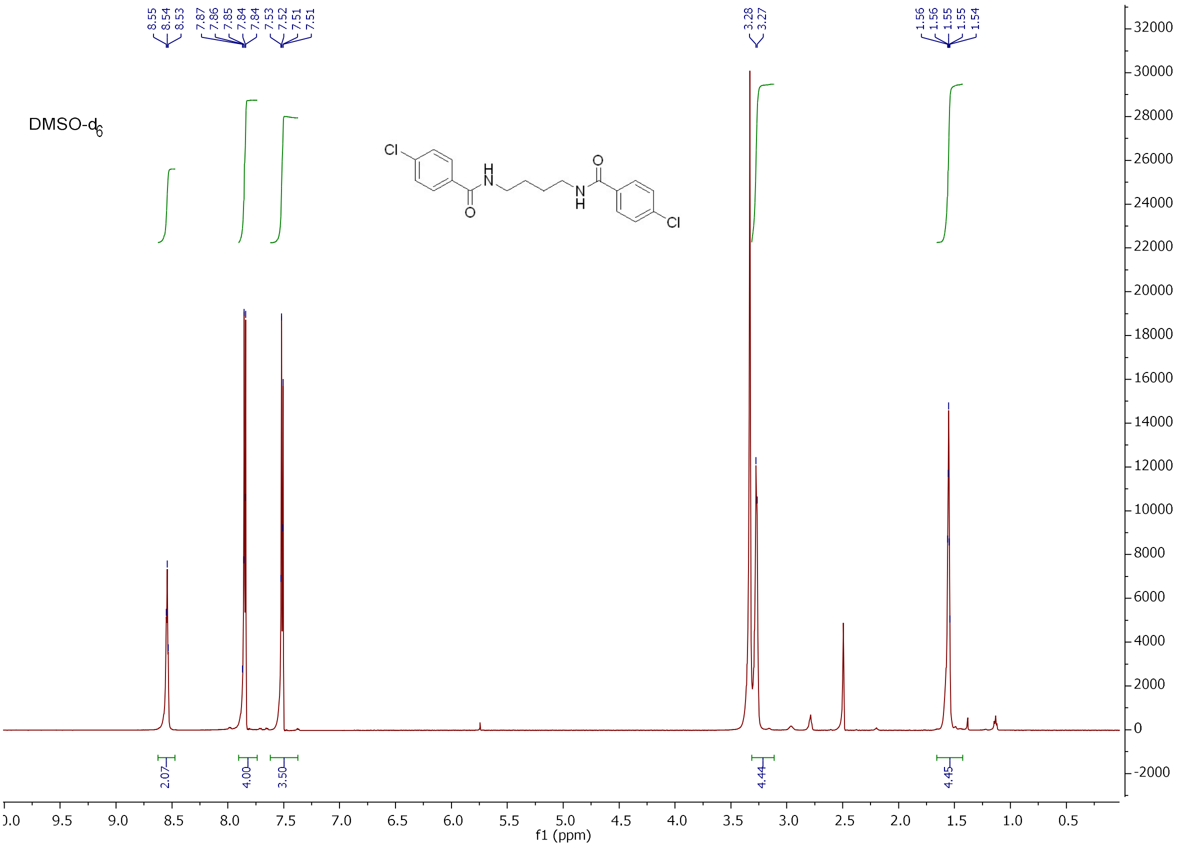

Supplementary Figure S28. [1]N-NMR **5c**.

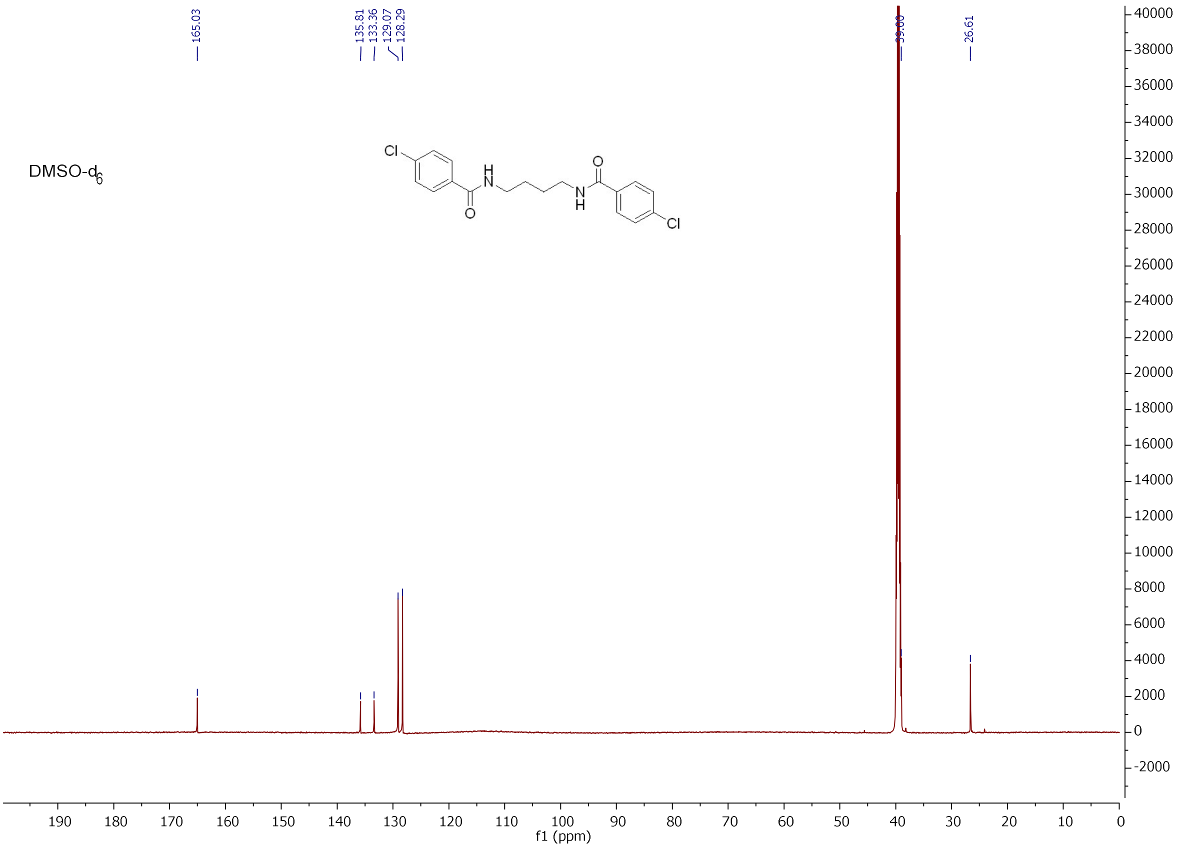

Supplementary Figure S29. ^{13}C-NMR **5c**.

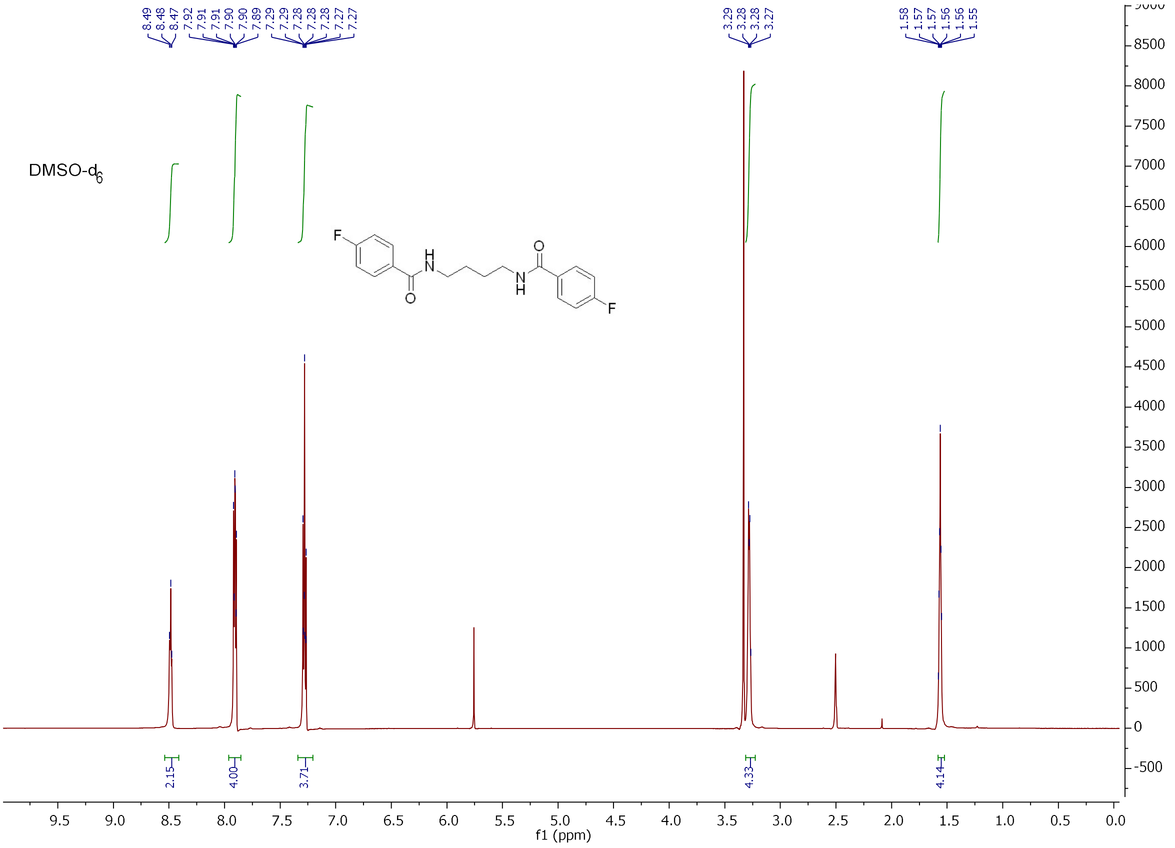

Supplementary Figure S30. ^{1}N-NMR **5d**.

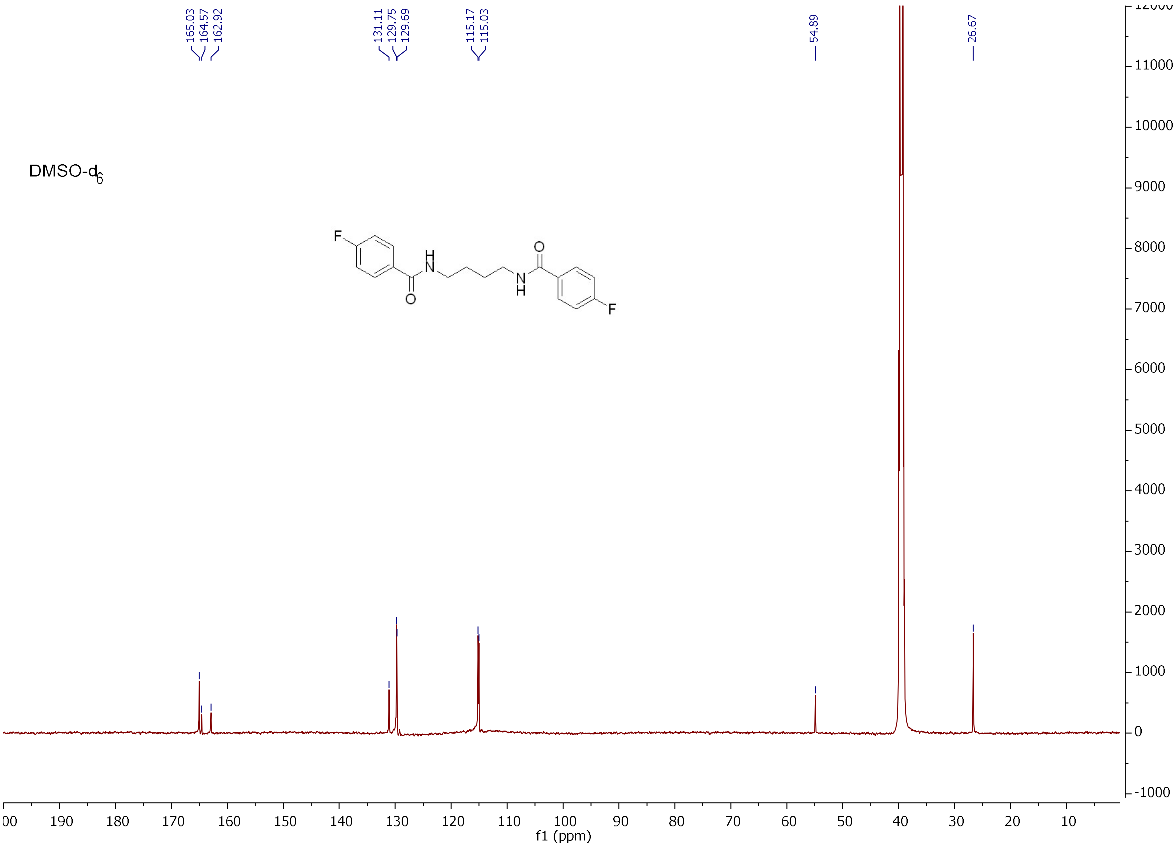

Supplementary Figure S31. ^{13}C-NMR 5**d**.

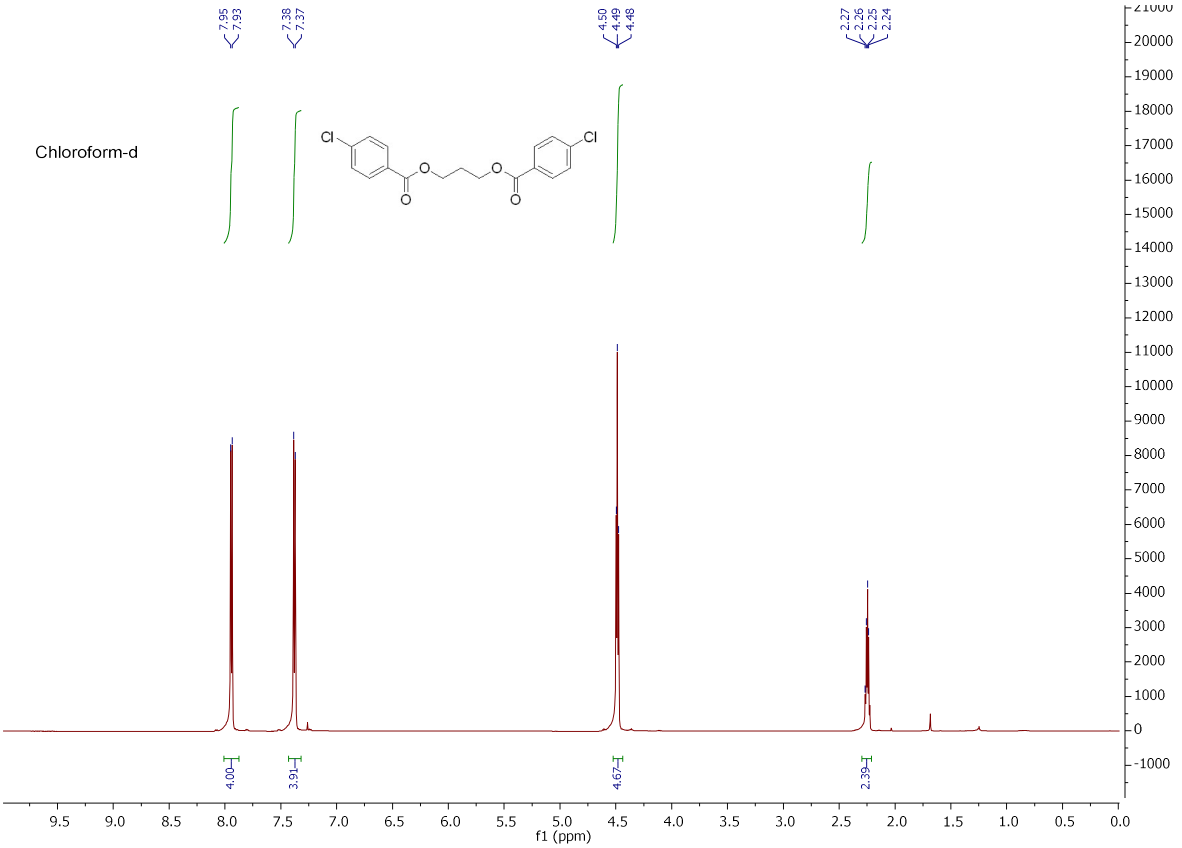

Supplementary Figure S32. ^{1}N-NMR **6a**.

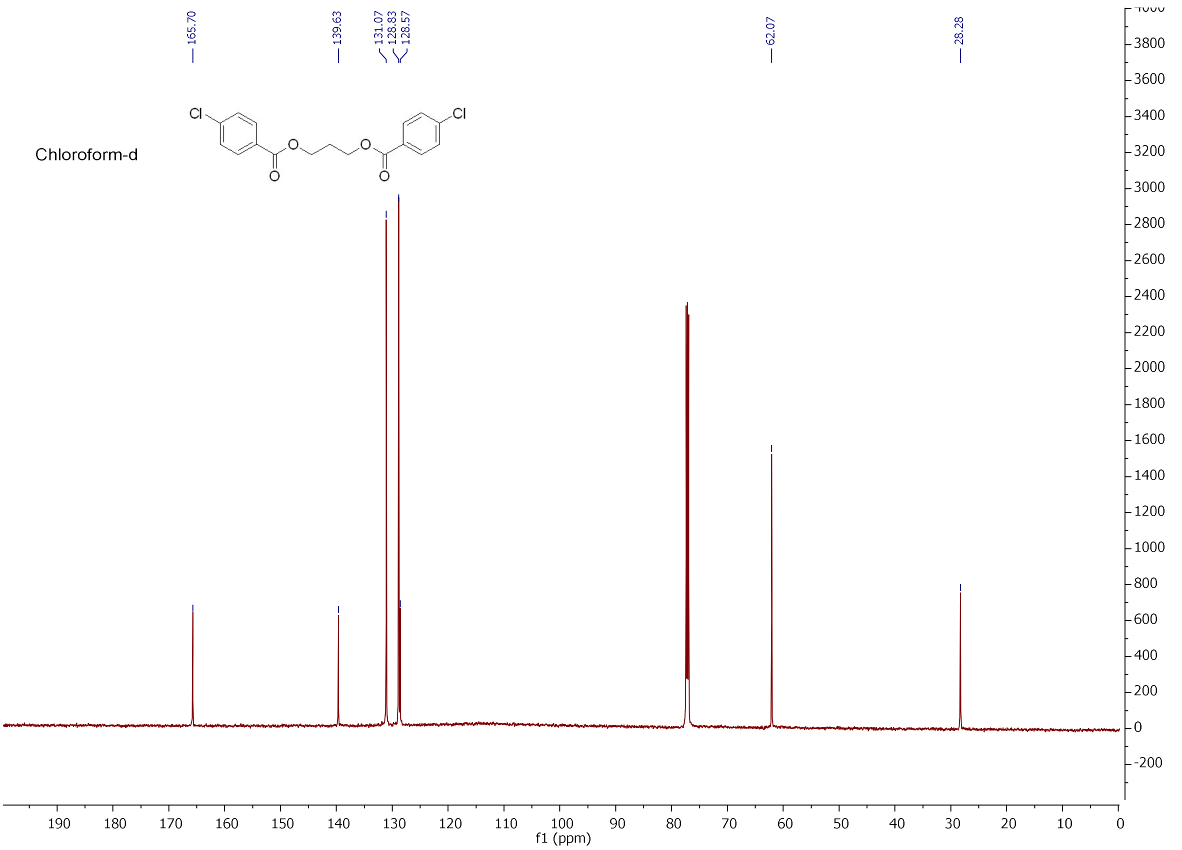

Supplementary Figure S33. ^{13}C-NMR **6a**.

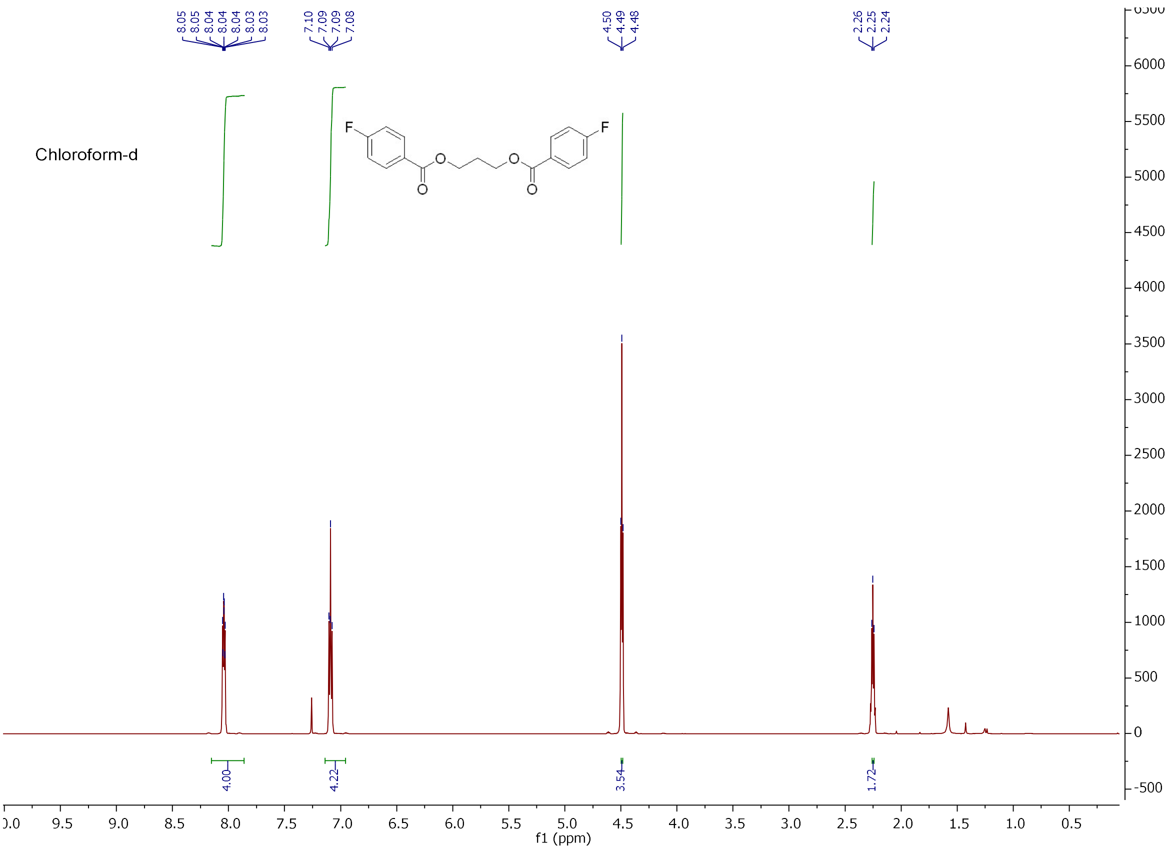

Supplementary Figure S34. [1]N-NMR **6b**.

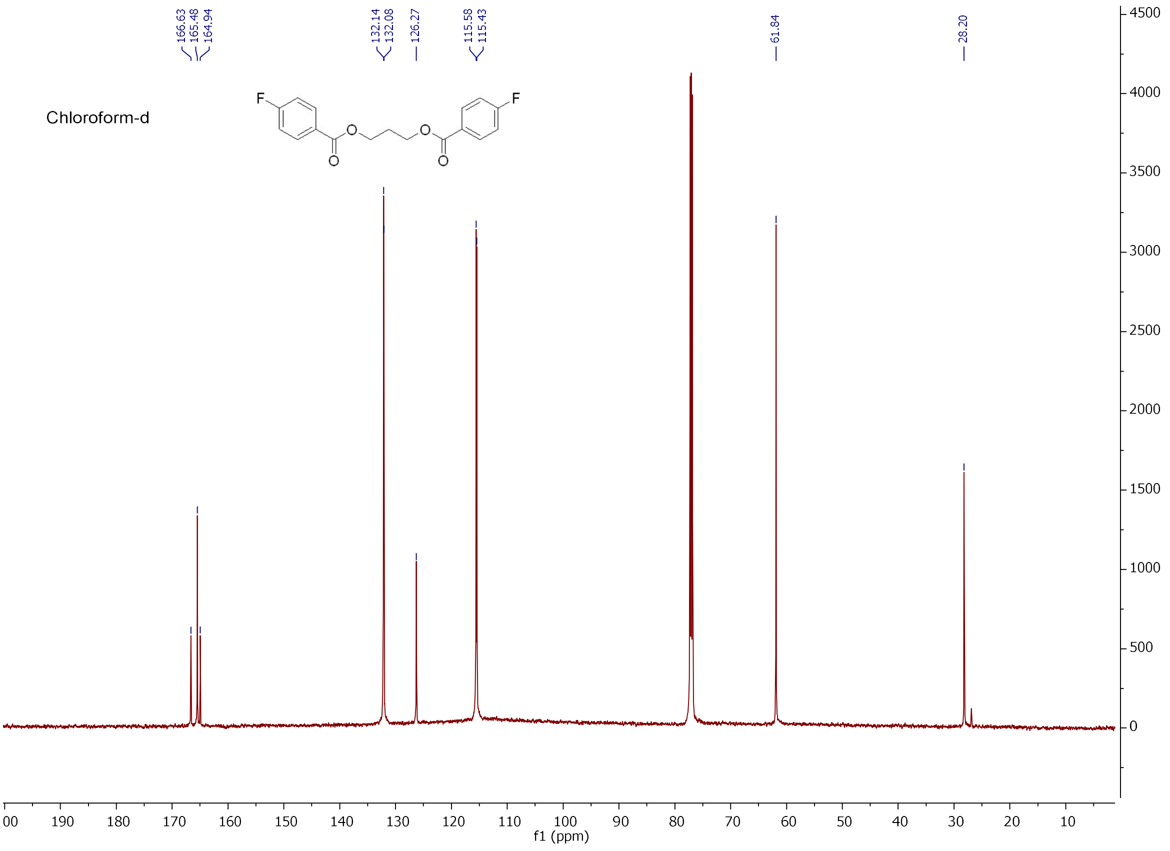

Supplementary Figure S35. ^{13}C-NMR **6b**.

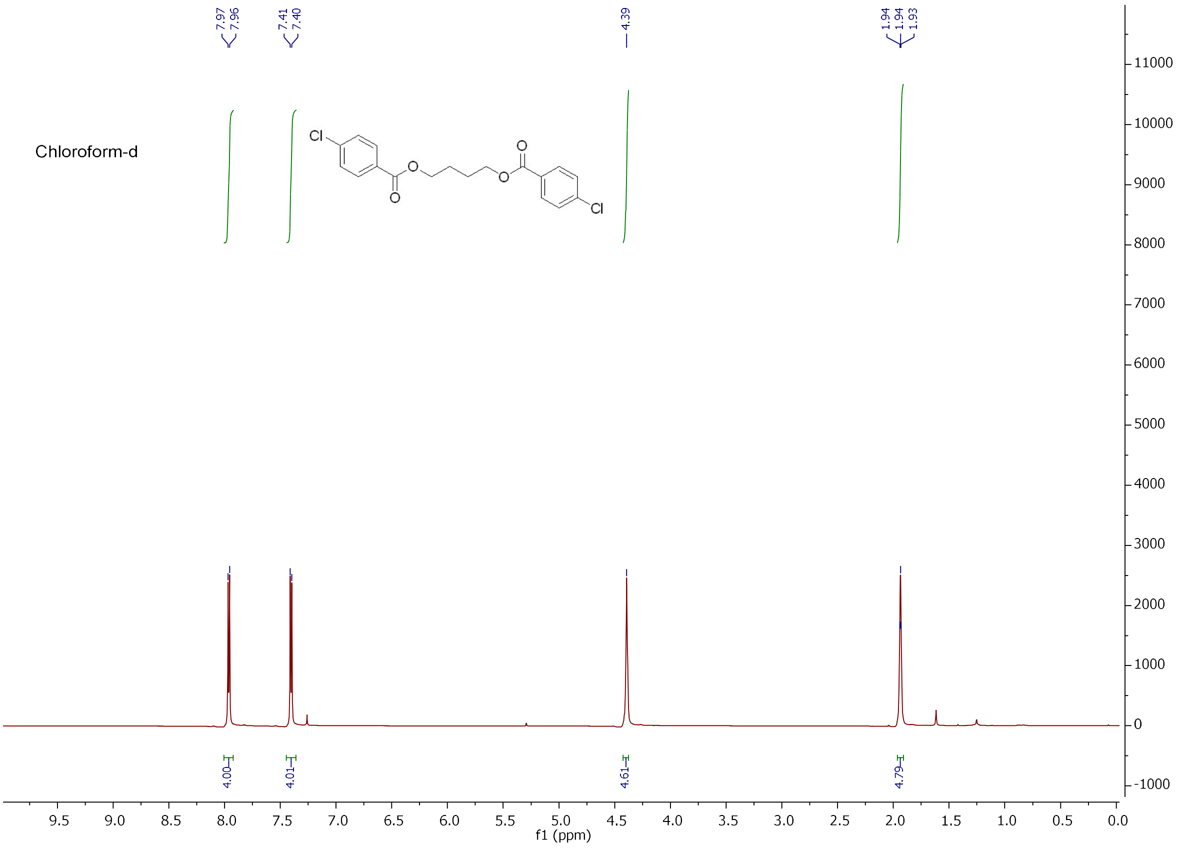

Supplementary Figure S36. [1]N-NMR **6c**.

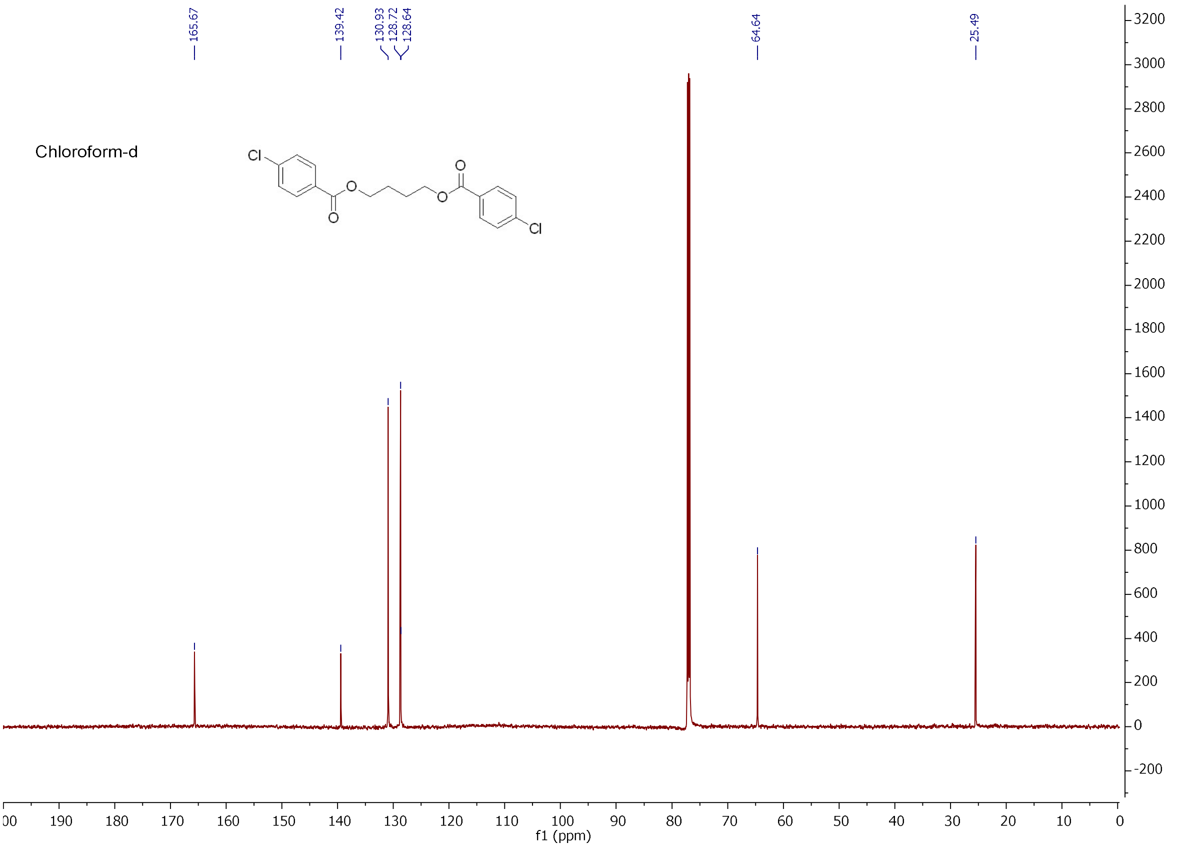

Supplementary Figure S37. ^{13}C-NMR **6c**.

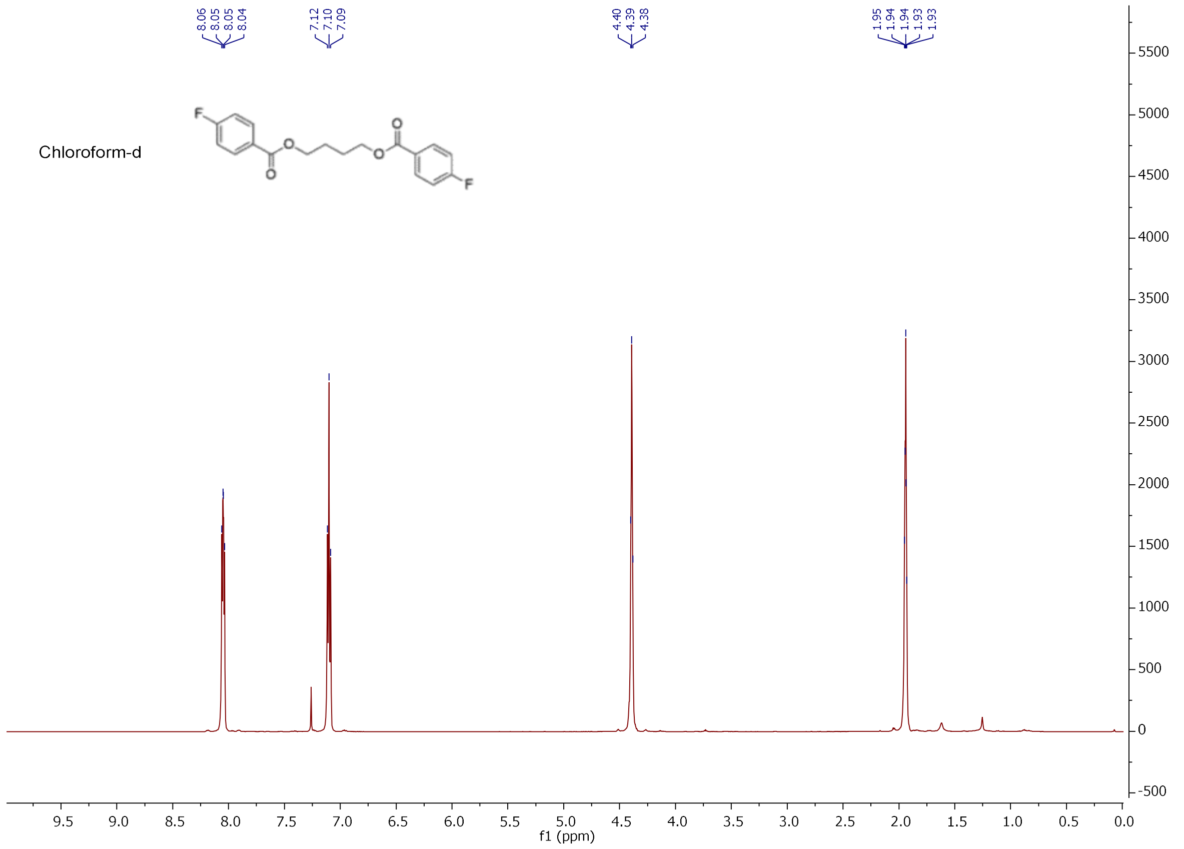

Supplementary Figure S38. ^{1}N-NMR **6d**.

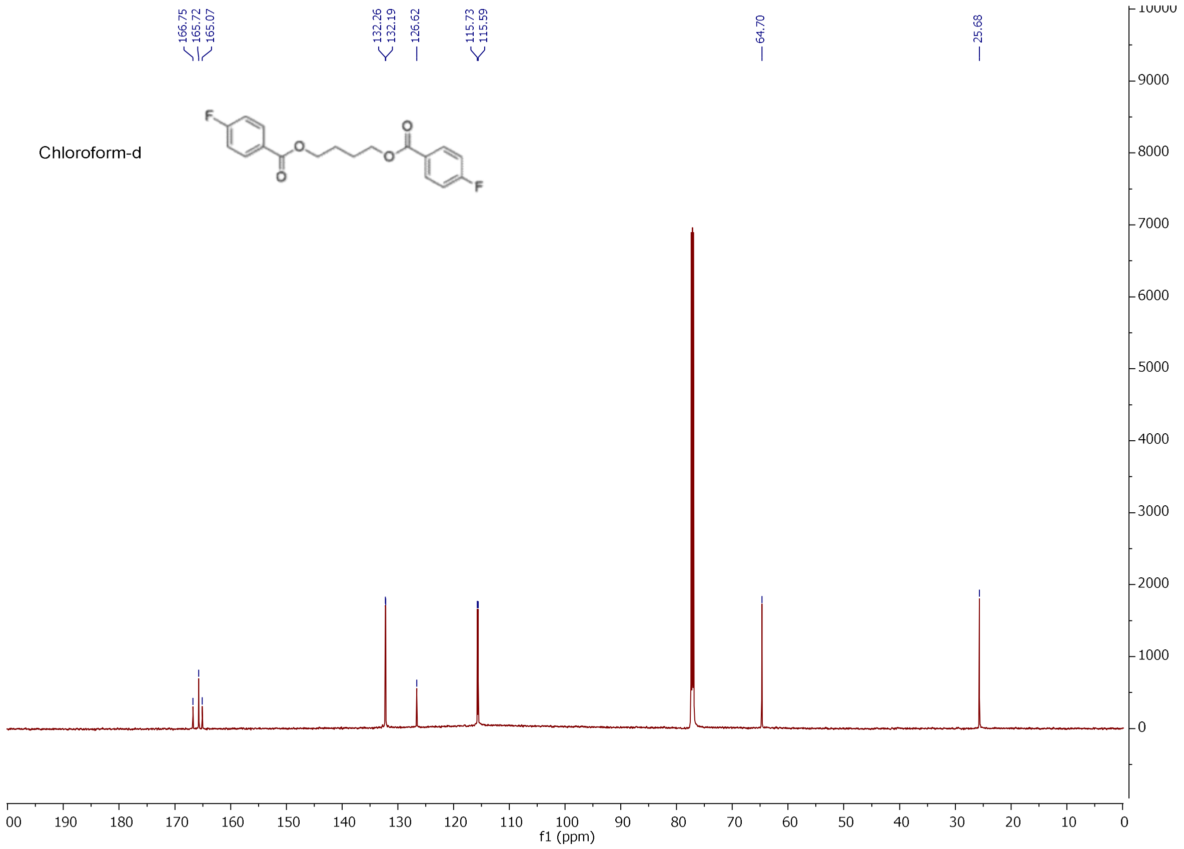

Supplementary Figure S39. ^{13}C-NMR **6d**.

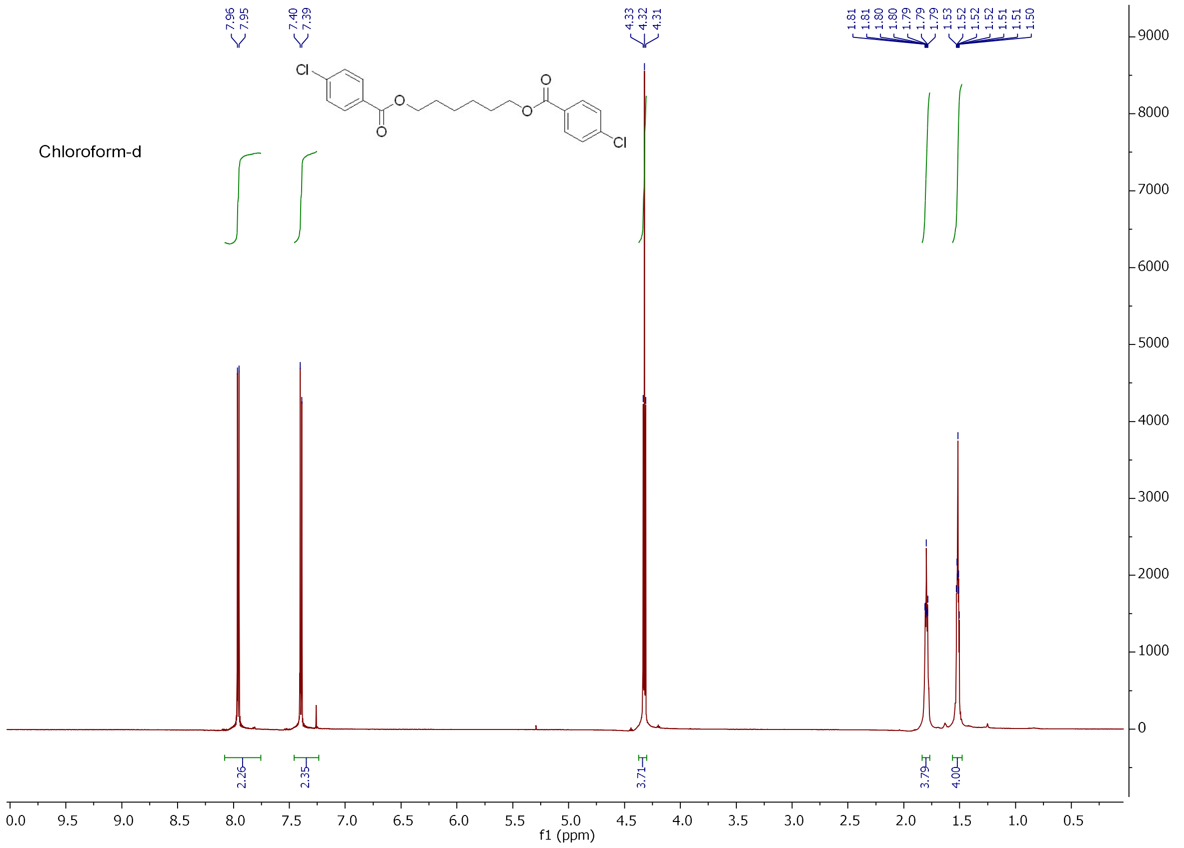

Supplementary Figure S40. [1]N-NMR **6e**.

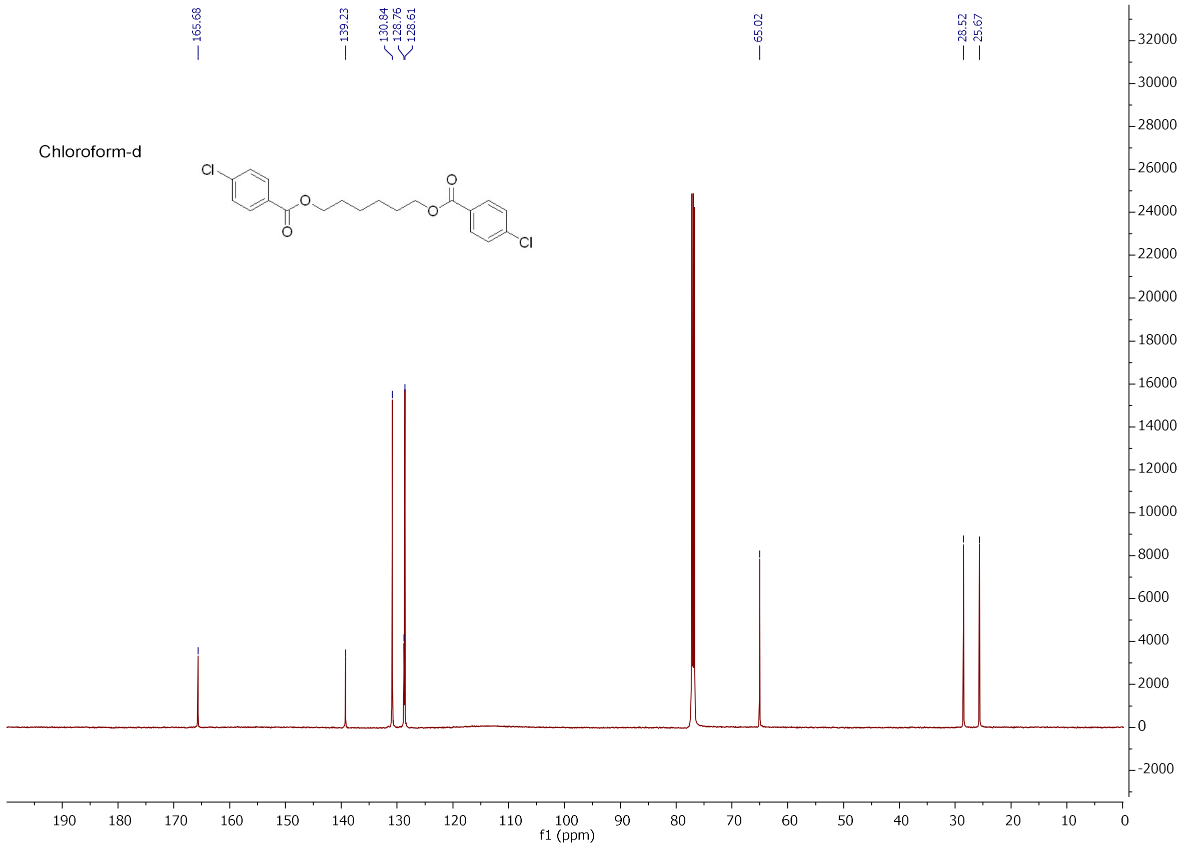

Supplementary Figure S41. ^{13}C-NMR **6e**.

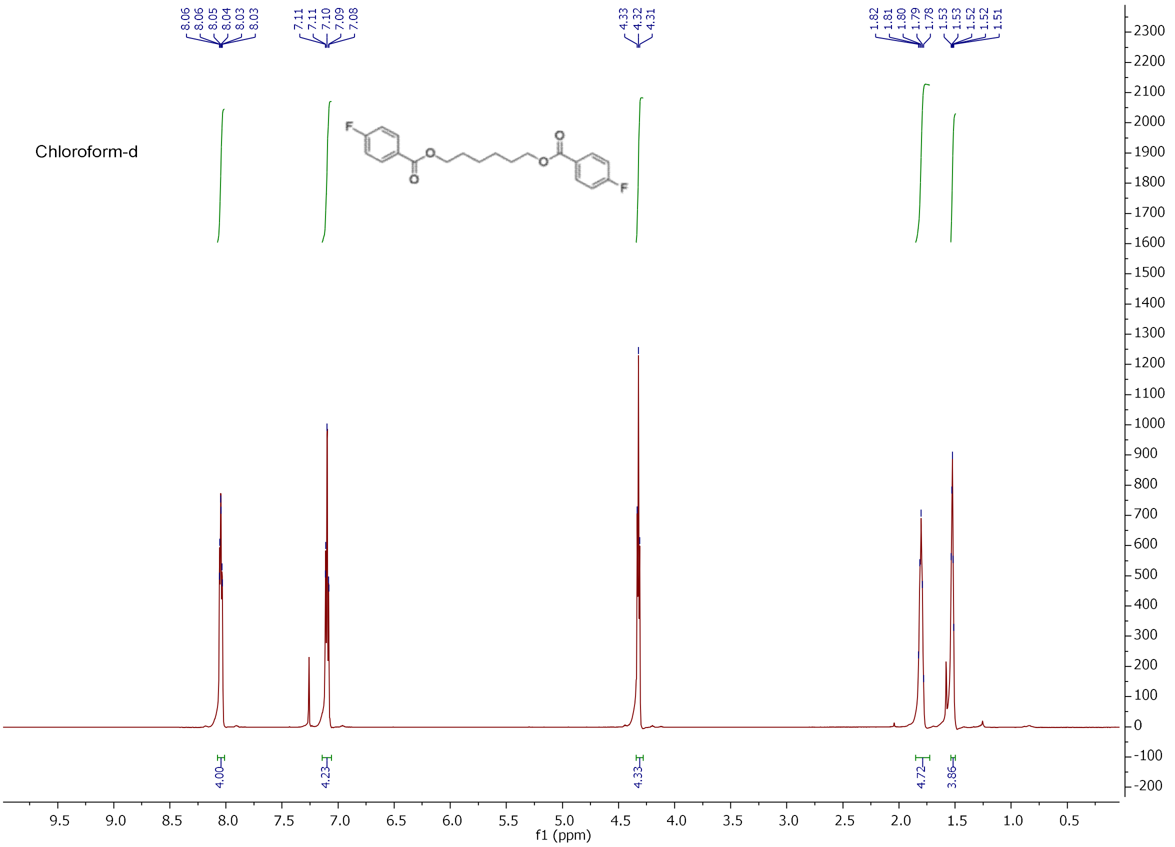

Supplementary Figure S42. ^{1}N-NMR **6f**.

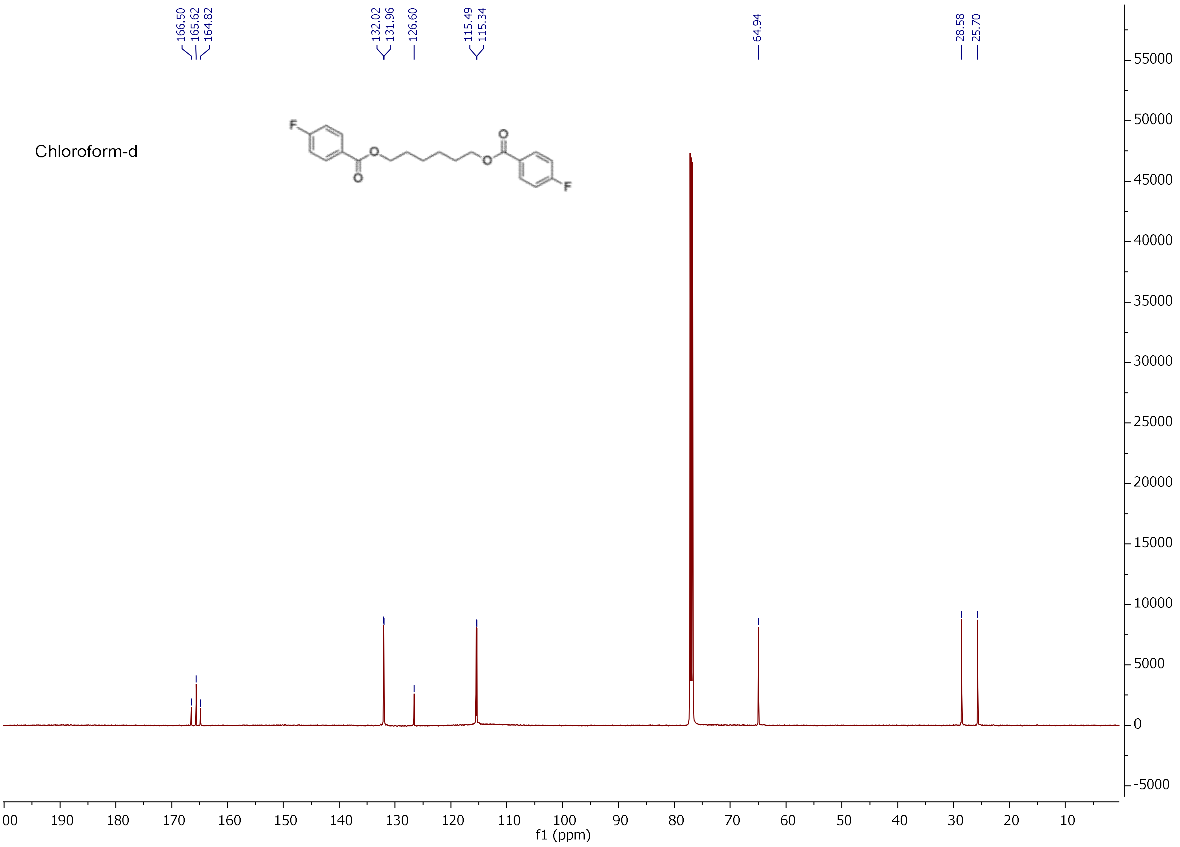

Supplementary Figure S43. ^{13}C-NMR **6f**.

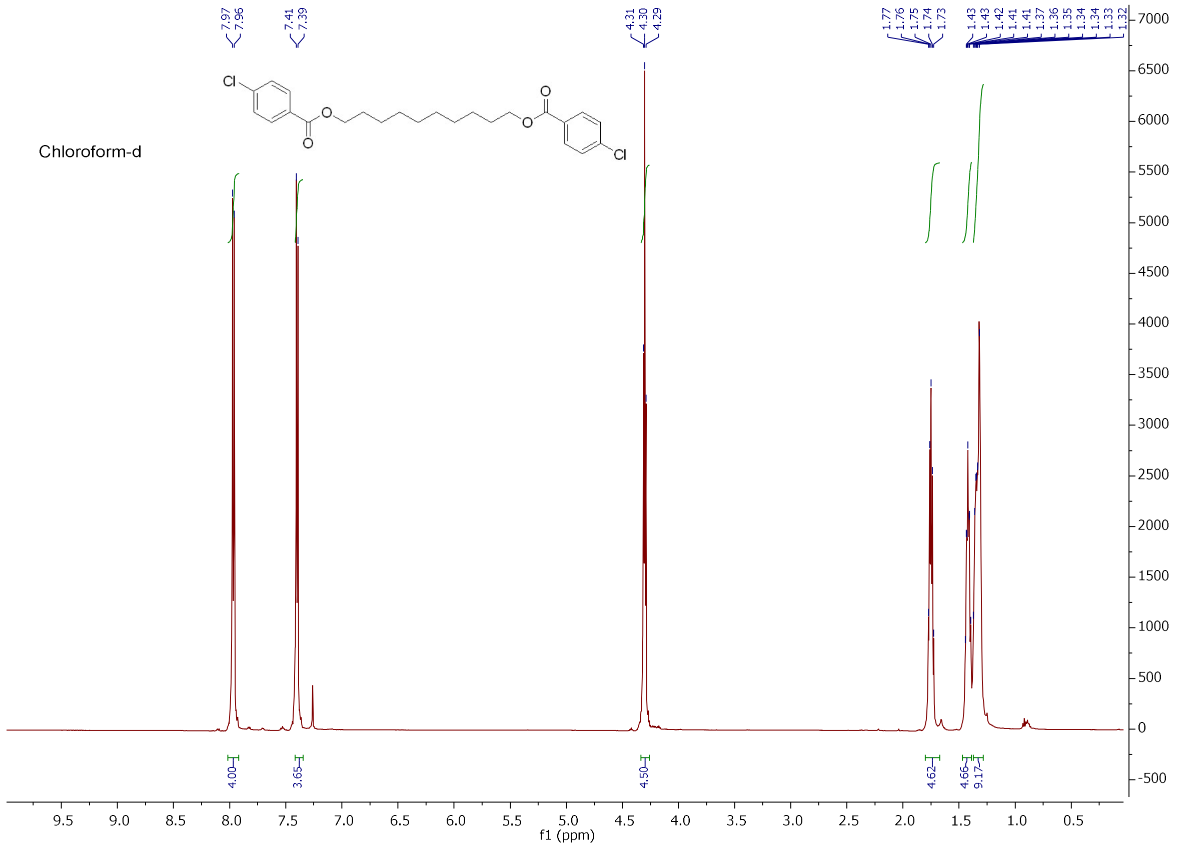

Supplementary Figure S44. [1]N-NMR **6g**.

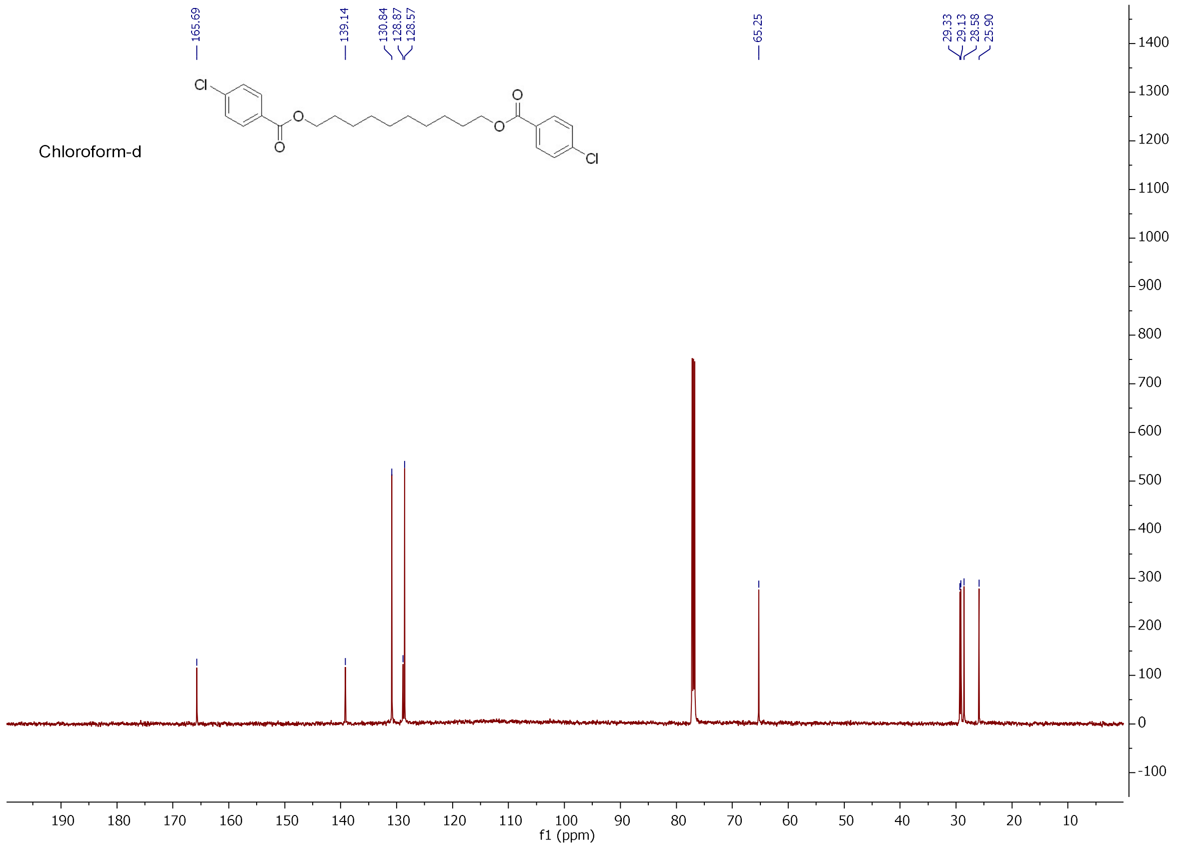

Supplementary Figure S45. ^{13}C-NMR **6g**.

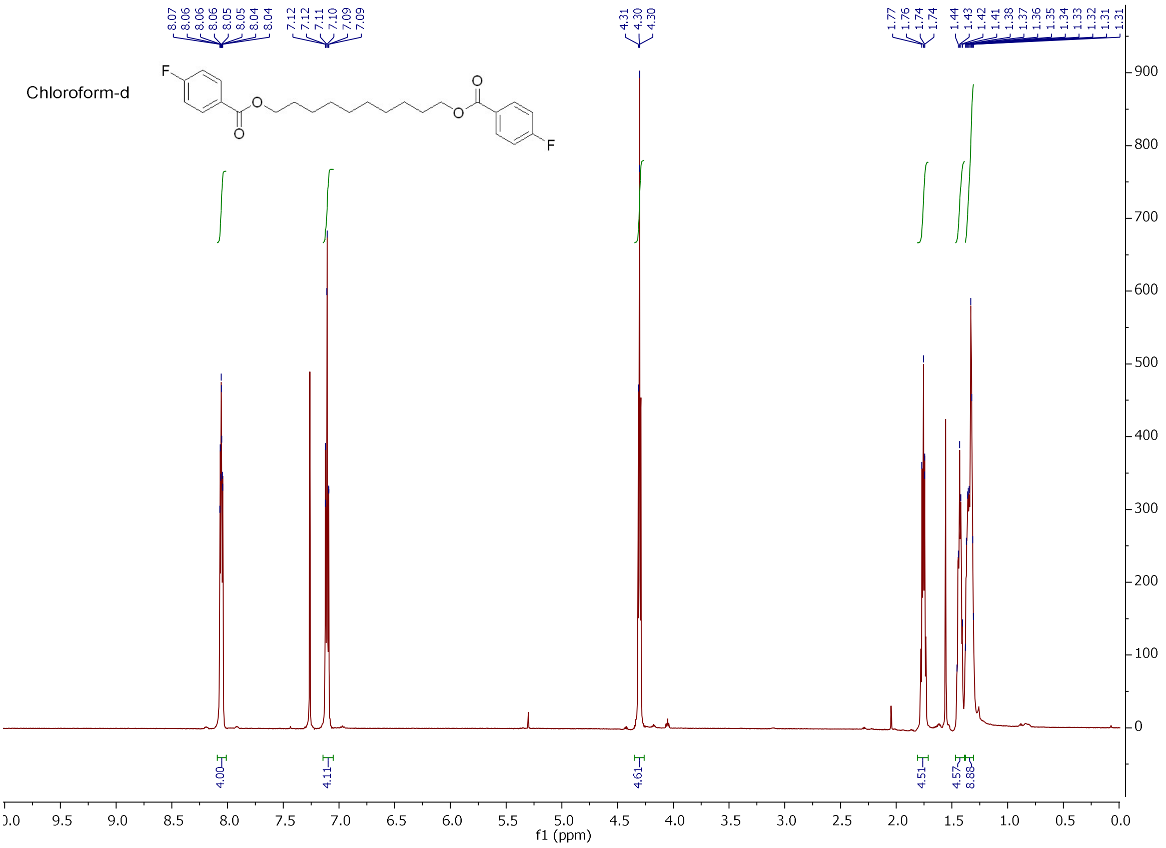

Supplementary Figure S46. ^{1}N-NMR **6h**.

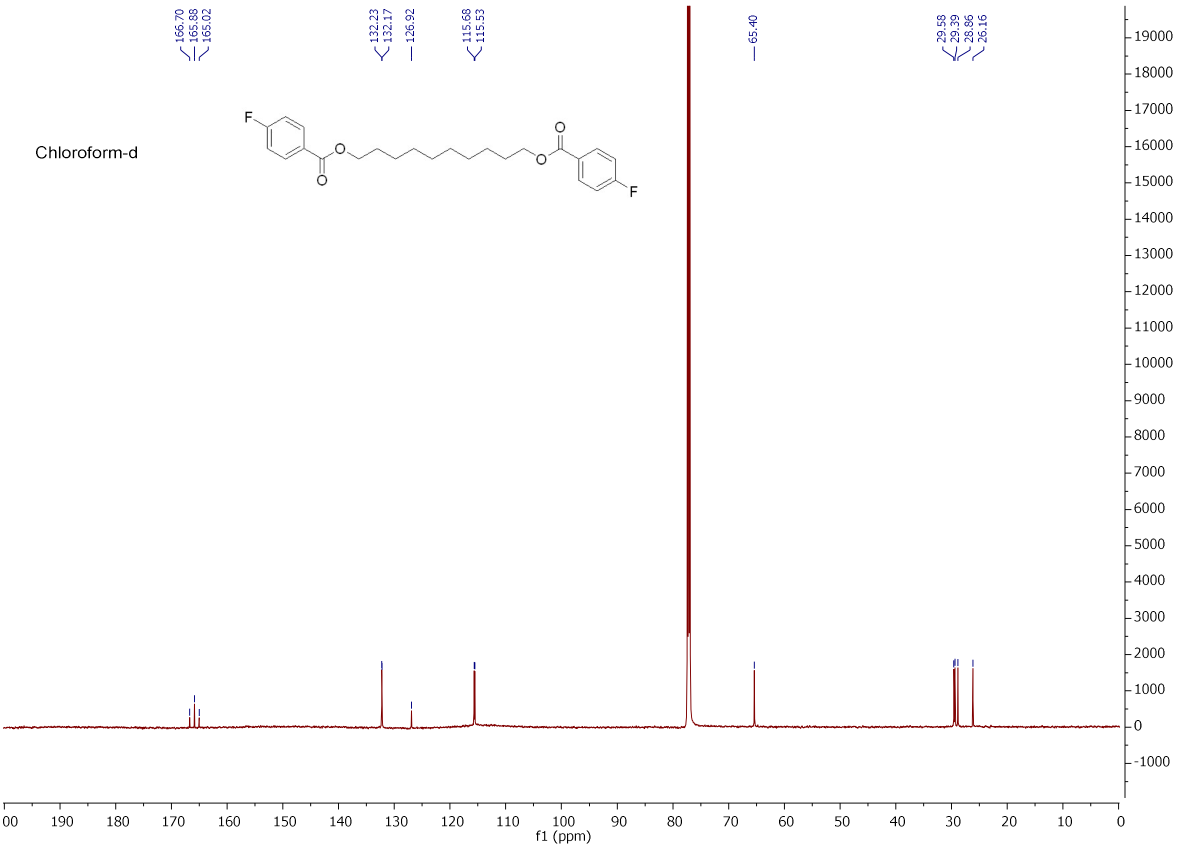

Supplementary Figure S47. ^{13}C-NMR **6h**.

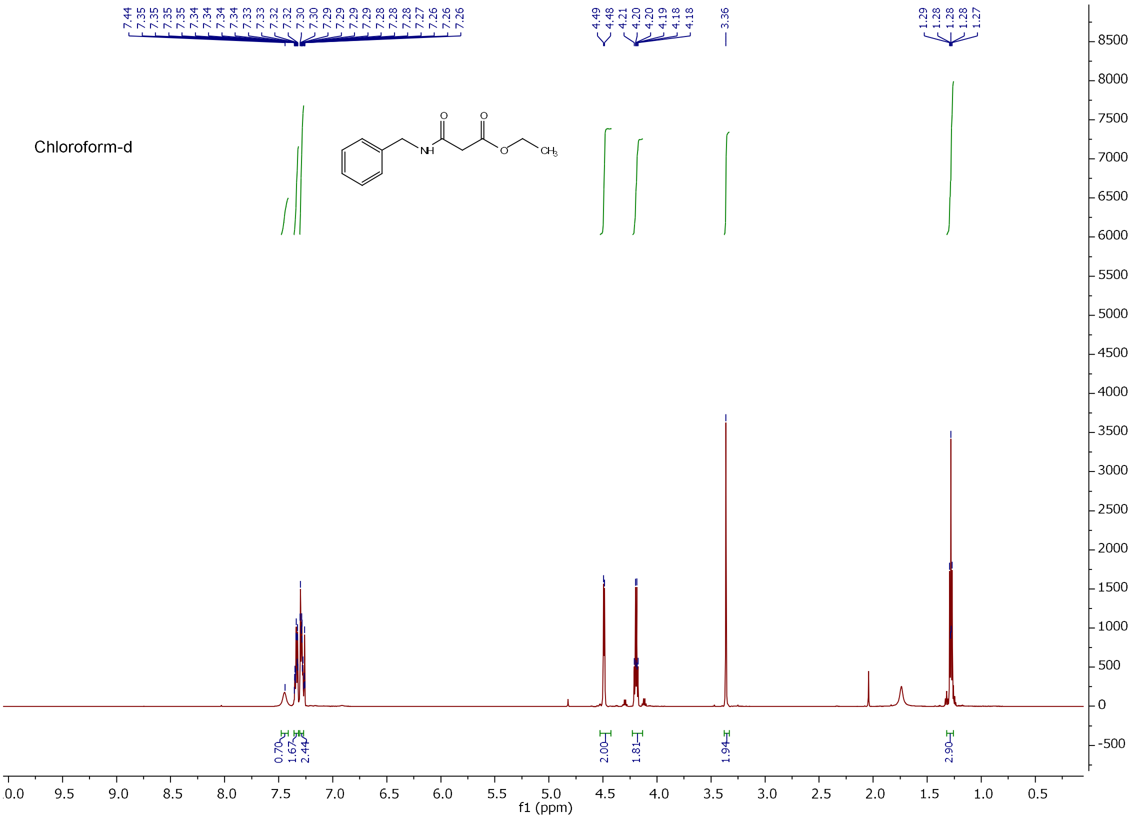

Supplementary Figure S48. ¹N-NMR **9**.

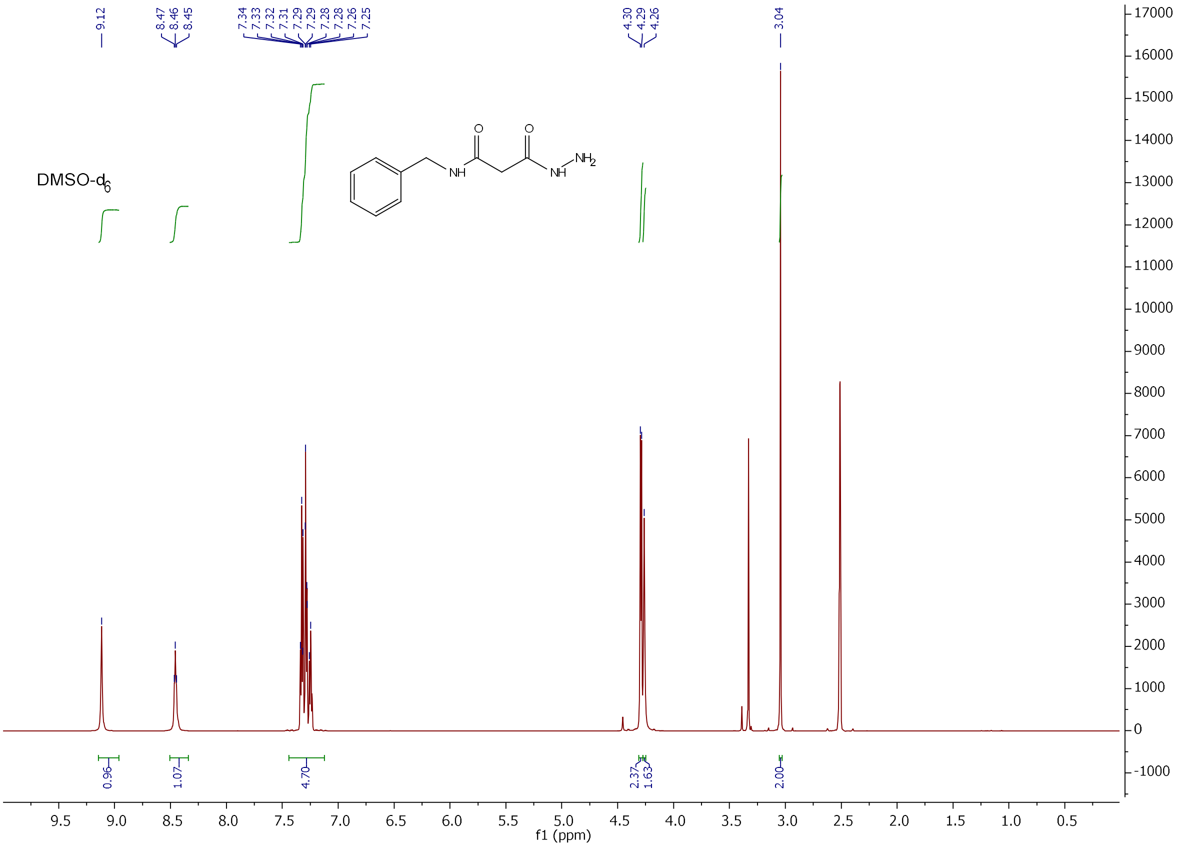

Supplementary Figure S49. ¹N-NMR **10**.

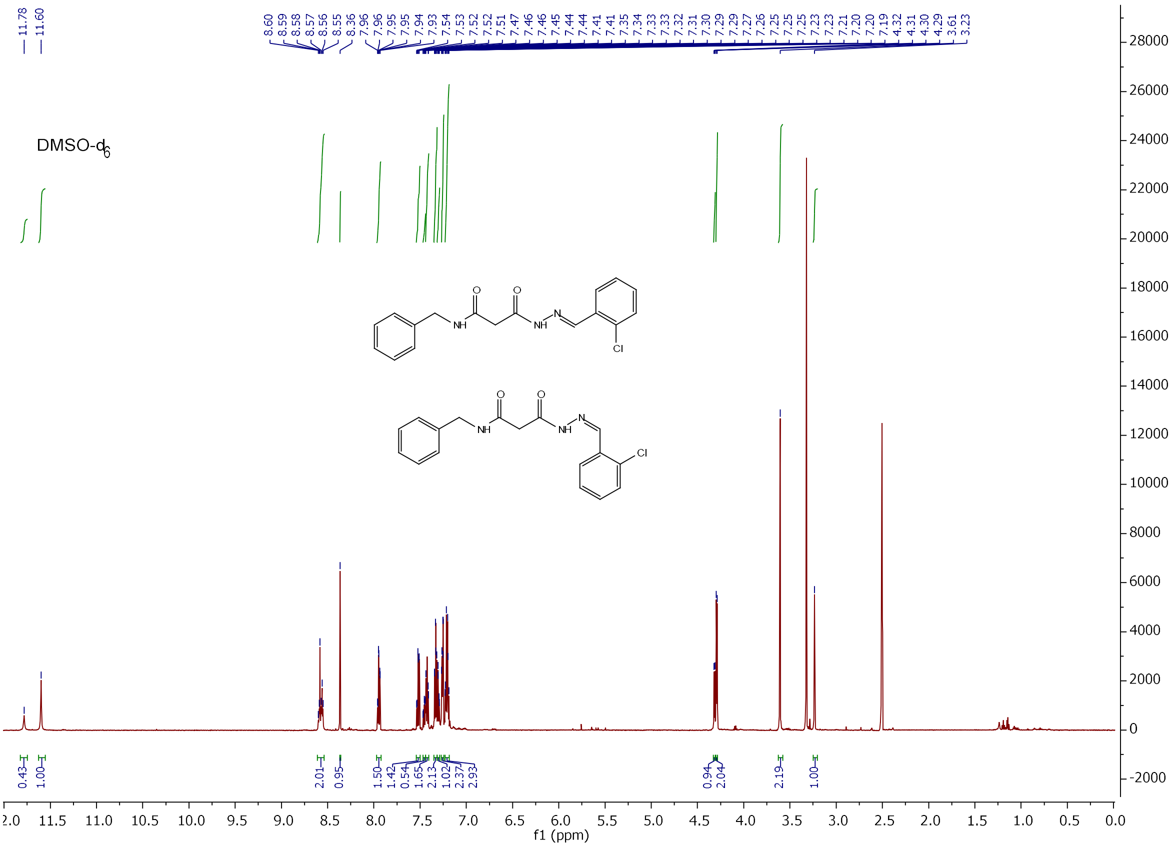

Supplementary Figure S50. [1]N-NMR **3**.

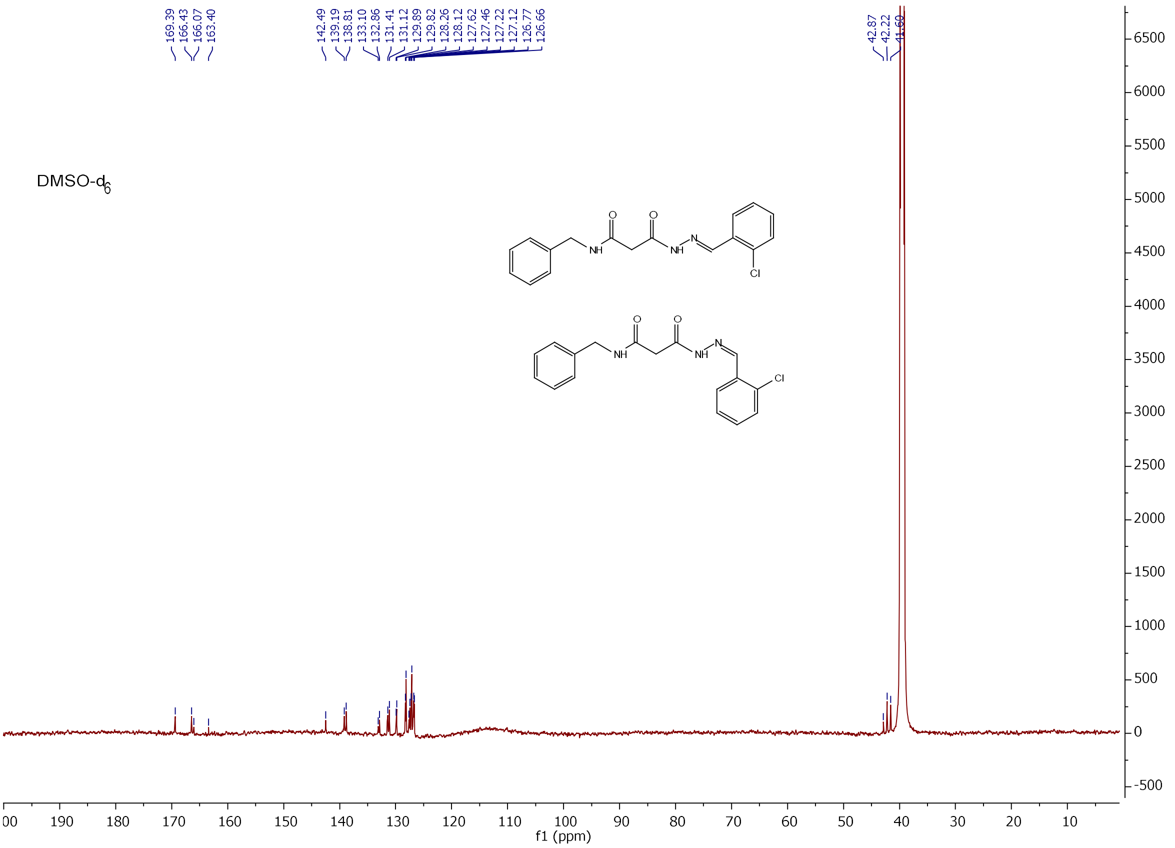

Supplementary Figure S51. ^{13}C-NMR **3**.
